# Alpha oscillatory activity is causally linked to working memory retention

**DOI:** 10.1371/journal.pbio.3001999

**Published:** 2023-02-13

**Authors:** Xueli Chen, Ru Ma, Wei Zhang, Ginger Qinghong Zeng, Qianying Wu, Ajiguli Yimiti, Xinzhao Xia, Jiangtian Cui, Qiongwei Liu, Xueer Meng, Junjie Bu, Qi Chen, Yu Pan, Nancy Xiaonan Yu, Shouyan Wang, Zhi-De Deng, Alexander T. Sack, Myles Mc Laughlin, Xiaochu Zhang

**Affiliations:** 1 Department of Radiology, the First Affiliated Hospital of USTC, Hefei National Research Center for Physical Sciences at the Microscale and School of Life Science, Division of Life Science and Medicine, University of Science & Technology of China, Hefei, China; 2 Department of Social and Behavioural Sciences, City University of Hong Kong, Hong Kong, People’s Republic of China; 3 Application Technology Center of Physical Therapy to Brain Disorders, Institute of Advanced Technology, University of Science & Technology of China, Hefei, China; 4 Division of Humanities and Social Sciences, California Institute of Technology, Pasadena, California, United States of America; 5 Centers for Biomedical Engineering, School of Information Science and Technology, University of Science & Technology of China, Hefei, China; 6 School of Optometry and Vision Science, Cardiff University, Cardiff, United Kingdom; 7 School of Biomedical Engineering, Anhui Medical University, Hefei, China; 8 School of Psychology, South China Normal University, Guangzhou, China; 9 Shanghai Key Laboratory of Brain-Machine Intelligence for Information Behavior, School of Business and Management, Shanghai International Studies University, Shanghai, China; 10 Institute of Science and Technology for Brain-inspired Intelligence, Fudan University, Shanghai, China; 11 Noninvasive Neuromodulation Unit, Experimental Therapeutics & Pathophysiology Branch, Intramural Research Program, National Institute of Mental Health, National Institutes of Health, Bethesda, USA; 12 Department of Cognitive Neuroscience, Faculty of Psychology and Neuroscience, Maastricht University, Maastricht, The Netherlands; 13 Exp ORL, Department of Neuroscience, Leuven Brain Institute, KU Leuven, Leuven, Belgium; 14 Department of Psychology, School of Humanities & Social Science, University of Science & Technology of China, Hefei, China; 15 Institute of Health and Medicine, Hefei Comprehensive National Science Center, Hefei, China; University of Glasgow, UNITED KINGDOM

## Abstract

Although previous studies have reported correlations between alpha oscillations and the “retention” subprocess of working memory (WM), causal evidence has been limited in human neuroscience due to the lack of delicate modulation of human brain oscillations. Conventional transcranial alternating current stimulation (tACS) is not suitable for demonstrating the causal evidence for parietal alpha oscillations in WM retention because of its inability to modulate brain oscillations within a short period (i.e., the retention subprocess). Here, we developed an online phase-corrected tACS system capable of precisely correcting for the phase differences between tACS and concurrent endogenous oscillations. This system permits the modulation of brain oscillations at the target stimulation frequency within a short stimulation period and is here applied to empirically demonstrate that parietal alpha oscillations causally relate to WM retention. Our experimental design included both in-phase and anti-phase alpha-tACS applied to participants during the retention subprocess of a modified Sternberg paradigm. Compared to in-phase alpha-tACS, anti-phase alpha-tACS decreased both WM performance and alpha activity. These findings strongly support a causal link between alpha oscillations and WM retention and illustrate the broad application prospects of phase-corrected tACS.

## Abstract 2

With its capacity to correct for its phase differences relative to concurrent brain oscillations, an online phase-corrected tACS system provides the possibility to both up- and down-regulate targeted brain oscillations within a short period; using such a system, this study shows that parietal alpha oscillations exert causal impacts on working memory retention.

## 1. Introduction

Working memory (WM) is considered to be foundational for a broad range of cognitive functions (e.g., the capacity for general intelligence, categorization, retrieving selected long-term memories, language learning) [1]. WM enables the maintenance, manipulation, and retrieval of mental representations, as well as the use of this information in goal-directed behaviors [1]. Because of its essential role in human cognition, investigating the neural mechanisms underlying WM has been a focus of neuroscience research for decades [2,3].

WM can be subdivided into 3 fundamental subprocesses: encoding, retention, and retrieval [4]. Neural oscillations in the alpha frequency band (8 to 13 Hz) have been associated with WM retention [5]: alpha activity in the occipitoparietal areas increases during memory retention [6–10]. Previous studies also reported that alpha power increased parametrically with memory load during the retention subprocess [6,11]. However, as the majority of these previous human studies only used MEG or electroencephalogram (EEG), their inferences are correlational in nature and cannot demonstrate a causal role for alpha oscillations in WM retention.

Transcranial alternating current stimulation (tACS), a means of noninvasive brain stimulation (NIBS), may provide us with the opportunity to experimentally investigate a causal role for alpha oscillations in WM retention, owing to its ability to entrain naturally occurring neural oscillations based on externally applied, sinusoidal electric fields at a targeted frequency [12–15]. It should be noted that results from previous tACS studies are at the center of a controversial debate in which some studies failed to replicate successful entrainment effects [16–18]. This discrepancy across tACS studies may be related to the fact that previous efforts have (to our understanding) not accounted for the phase of ongoing brain oscillations [16]. Previous phase-locked repetitive transcranial magnetic stimulation (rTMS) studies have reported that the phase of ongoing brain oscillations at the moment of rTMS application impacts the efficacy of stimulation effects [19,20]. In tACS studies, recent computational model [21], animal study [22], and indirect experimental evidence correcting for the phase differences between tACS and peripheral tremor in Parkinson’s patients [23] also suggest that the phase differences between tACS and concurrent brain oscillations are impactful for determining the efficacy of tACS (also for determining the robustness of effects and replicability) [21]. But to our knowledge, any direct experimental evidence based on correcting for the phase differences between tACS waveforms and brain oscillations in humans is absent.

Moreover, contrary to the retention subprocess, decreased (rather than increased) alpha oscillations during the encoding and retrieval subprocesses were reported to be beneficial for WM performance [24,25]. Thus, tACS investigating the causal role for alpha oscillations in WM retention should be time-locked to the specific WM subprocess of interest (i.e., retention but not encoding or retrieval), to avoid the potential offset effects of conventional tACS applied during the other 2 subprocesses given that conventional tACS was usually applied continuously for 20 to 30 min.

Given this background, there are 2 major reasons that conventional tACS methods do not support experimental investigations about the function(s) of alpha oscillations in WM retention: conventional tACS methods cannot correct for phase differences between tACS waveforms and brain oscillations in real-time (“online”), and conventional tACS methods do not support tACS stimulation that can be time-locked to the specific short-time subprocess of WM. These ideas motivated our desire for a tACS technology with the following capabilities: (1) a capacity for real-time monitoring of endogenous alpha oscillatory activity, to support real-time phase alignment between externally applied tACS and concurrent endogenous alpha oscillations; and (2) a capacity to induce short-duration (within seconds) tACS in a time-locked manner that can be matched to the short-duration retention subprocess in each trial.

**Fig 1:**
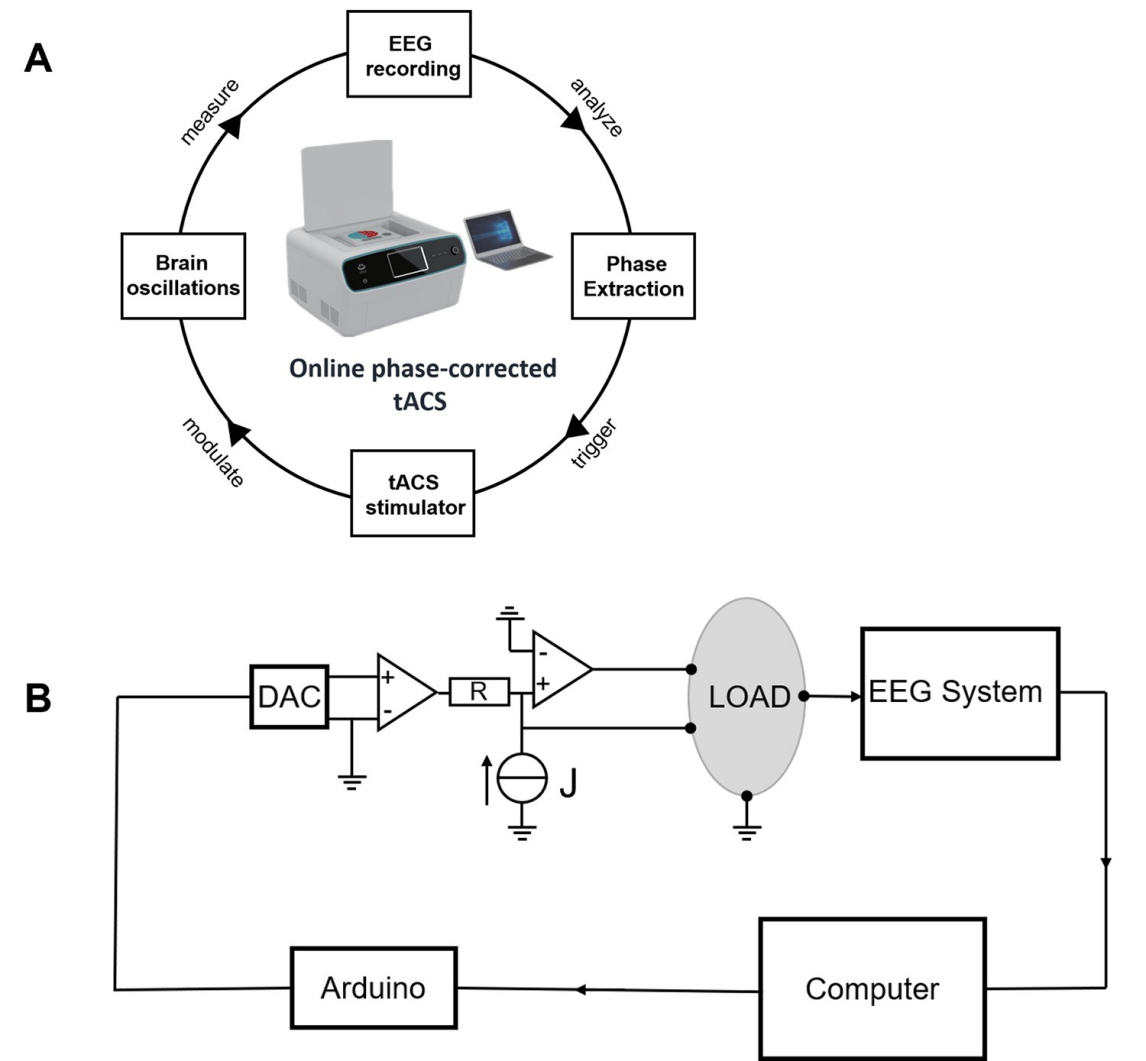
The components of the online phase-corrected tACS system and the design of the tACS stimulator. (**A**) The online phase-corrected tACS system consists of an EEG instrument measuring brain oscillations, a computer extracting the real-time phases of brain oscillations to decide the timing of the tACS stimulator, and a custom-designed tACS stimulator that communicates with the computer to regulate the application of tACS to the human brain. (**B**) The design of the tACS stimulator. The major components of the tACS stimulator include an Arduino Uno microcontroller board, a DAC, a constant-voltage source (J), and 2 operational amplifiers. The appropriate time point to initiate the stimulation (calculated from the computer) is transmitted to the Arduino board. The triangle represents the operational amplifier; R represents the resistance. DAC, digital-to-analog converter; EEG, electroencephalogram; tACS, transcranial alternating current stimulation.

**Fig 2:**
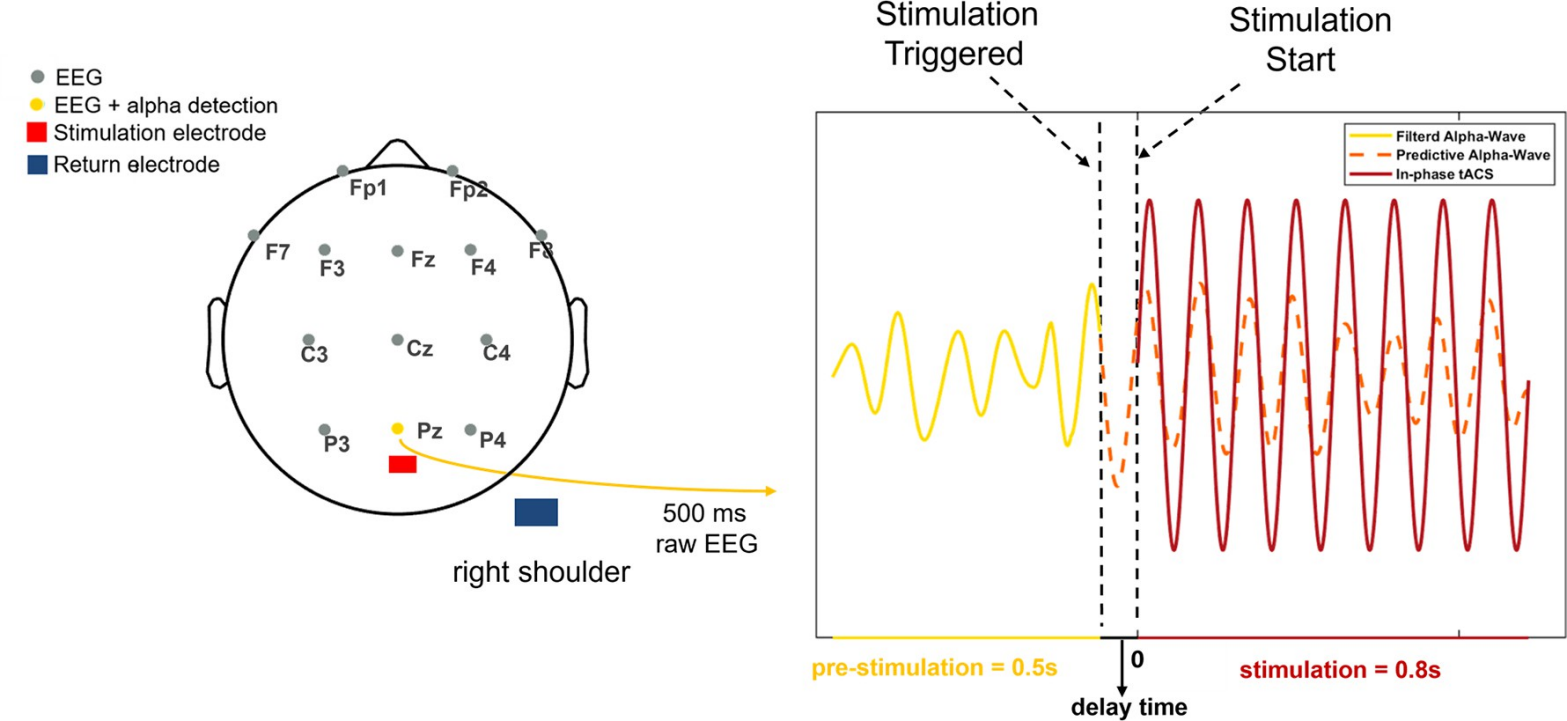
Illustration of the online phase-corrected tACS system. The left panel shows a standard 10–20 electrode system used in the experiment; the right panel shows an example of alpha-wave detection and the application of in-phase tACS. The stimulation electrode (shown with the red rectangle in the left panel) was placed over the central parietal-occipital cortex (between the Pz electrode and Oz electrode), with the return electrode over the right shoulder (shown with the blue rectangle in the left panel). The raw EEG data at the retention subprocess of the Sternberg task, as measured from the Pz electrode, was stored in a moving time window of 500 ms with a 10 ms step. This signal was filtered within the frequency range of IAF ± 2 Hz (shown with the dark yellow solid line) in real time. When 2 consecutive peaks exceeding the threshold were detected (note that the threshold was determined using Baseline EEG data), stimulation was triggered; subsequently after a specific delay time, in-phase tACS (shown in dark red) was initiated and lasted for 0.8 s. The dark orange dashed line shows the alpha wave after the triggering of stimulation (please note that it is not recorded due to tACS artifacts). The illustrative waveforms in the figure do not represent the actual size. EEG, electroencephalogram; IAF, individual alpha frequency; tACS, transcranial alternating current stimulation.

**Fig 3:**
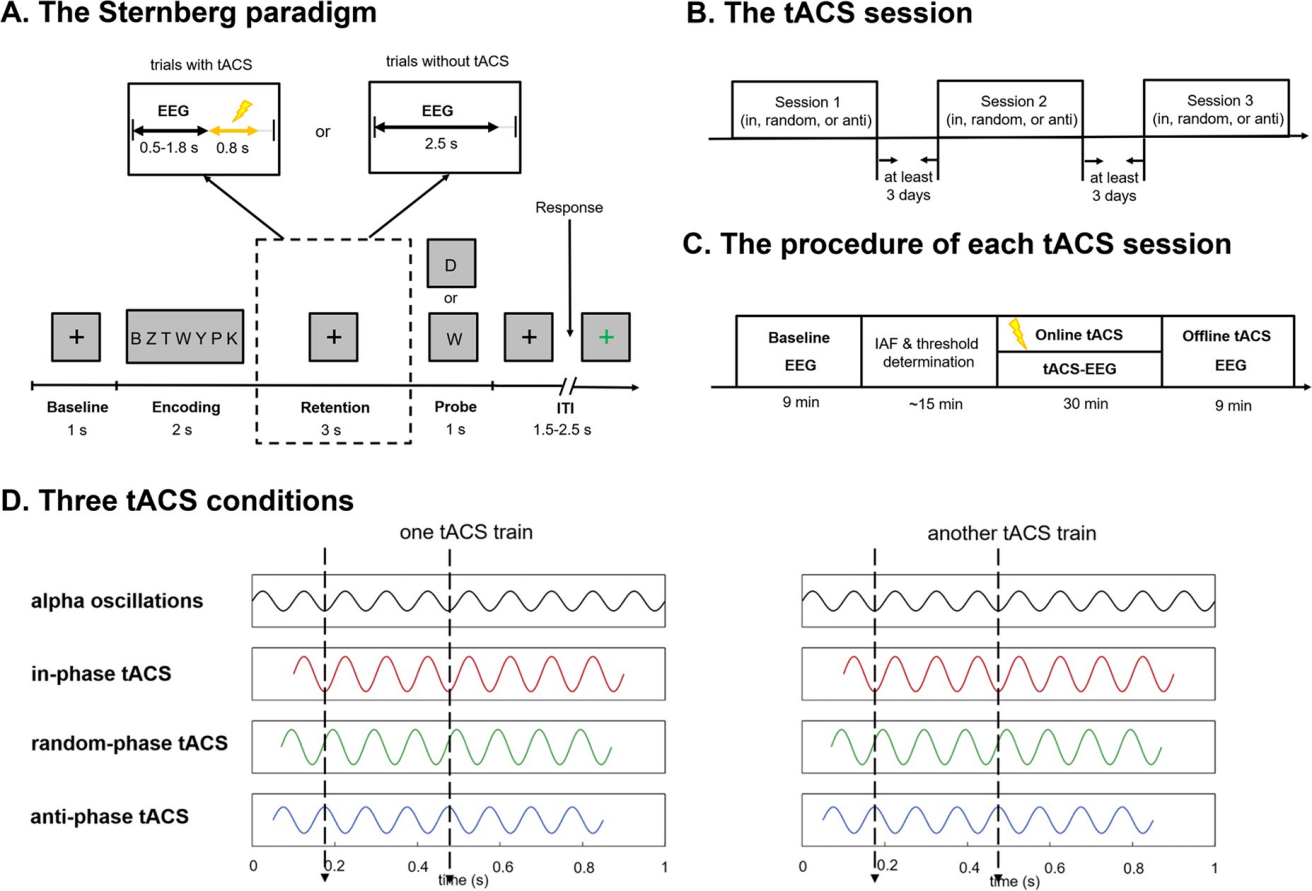
The designs of Experiment 1. (**A**) Schematic representation of the modified Sternberg paradigm used in this study. For each trial, participants were shown a list of 7 consonants and were asked to indicate (by button press) whether the probe was part of the memory list. For trials with tACS application, EEG recording started from the onset of the retention subprocess, and stopped once tACS was triggered; for trials where tACS was not triggered, EEG recording lasted for 2.5 s since the beginning of the retention subprocess. (**B**) Participants completed 3 tACS sessions of different conditions (in-phase tACS, random-phase tACS, or anti-phase tACS). The order of the tACS conditions was counterbalanced across participants, and the 3 sessions of each participant were all separated by at least 3 days. (**C**) Experimental procedure for each session. Within each session, participants first completed 60 trials of the Sternberg task with EEG recorded during the retention subprocesses (“Baseline”). Subsequently, the IAF of this session was calculated using the Baseline EEG data. Then, 180 tACS-EEG trials of the Sternberg task were performed with tACS delivered specifically during the retention subprocesses (“Online tACS”). After Online tACS, participants completed 60 trials of the Sternberg task, while EEG data were recorded during the retention subprocesses (“Offline tACS”). (**D**) tACS was delivered in 3 different conditions: (1) in the in-phase condition, tACS was applied at the IAF with 0° relative phase to the endogenous alpha oscillations; (2) in the anti-phase condition, tACS was applied at the IAF with 180° relative phase to the endogenous alpha oscillations; and (3) in the random-phase condition, no phase alignment between the delivered tACS and the detected endogenous alpha oscillations was used, with the phase differences changing from 0° to 360° across trials. For all conditions, the onset phase of the sine-wave tACS waveform was invariably 0°. The illustrative waveforms in the figure do not represent the actual sizes. EEG, electroencephalogram; IAF, individual alpha frequency; tACS, transcranial alternating current stimulation.

The present study successfully developed a trial-by-trial tACS-EEG design that is capable of correcting for the phase differences between applied tACS waveforms and concurrent endogenous oscillations (**Figs 1 and 2**), specifically during the retention subprocess of a Sternberg WM task [26]. Importantly, this technique allowed us to directly investigate whether alpha oscillations exert any causal impacts on WM retention. In Experiment 1, participants received parietal alpha tACS with 3 distinct phase differences relative to the real-time monitored endogenous alpha oscillations (in-phase tACS, anti-phase tACS, or random-phase tACS, **Fig 3D**) applied specifically during the WM retention subprocess. Fascinatingly, we found that compared to in-phase alpha-tACS, anti-phase alpha-tACS suppressed WM performance, parietal alpha power, and frontoparietal alpha synchronization. We also noted that changes in WM performance induced by in-phase alpha-tACS were positively correlated with changes in endogenous alpha oscillatory activity. We subsequently conducted Experiment 2 with a new cohort of participants; Experiment 2 successfully replicated the principle behavioral and electrophysiological findings of Experiment 1. Finally, we conducted a theta control experiment (Experiment 3) to exclude any general effects of tACS (i.e., independent of frequency) [27] and a phosphene control experiment (Experiment 4) to exclude possible impacts from retinal entrainment. Ultimately, beyond experimentally demonstrating a causal role for parietal alpha oscillations in WM retention, our results clearly illustrate how phase differences between tACS and concurrent endogenous brain oscillations determine the efficacy and replicability of tACS effects.

## 2. Results

In Experiment 1, we used the online phase-corrected tACS system to modulate alpha oscillations during the retention subprocess of WM (**Figs 1 and 2**; Materials and methods; S1 Text: “1.1. The details of online phase-corrected tACS system”), while participants performed a Sternberg task (**Fig 3A**; Materials and methods) [26]. tACS with a 2-mA peak-to-peak amplitude at individual alpha frequency (IAF, see S1 Text: “1.2. IAF and threshold determination”) was applied to the central parietal-occipital brain areas in in-phase, anti-phase, or random-phase conditions (**Fig 3D**; Materials and methods). Each participant performed a session of each tACS condition (in-phase tACS, anti-phase tACS, or random-phase tACS) separated by at least 3 days (**Fig 3B**). The experimental procedures for the 3 sessions are the same, except for the tACS condition (**Fig 3C**). Before examining tACS effects, we assessed the phase accuracy of the online phase-corrected tACS system. We analyzed the phase alignment between unstimulated EEG signals recorded at Baseline and artificial tACS waveforms generated offline; the results supported the phase accuracy of the tACS system (**S2 Fig**).

### 2.1. No systematic differences between in-phase tACS and anti-phase tACS at Baseline

In order to test the main hypothesis that tACS effects depend on the phase differences between tACS and endogenous brain oscillations, the different effects between in-phase tACS and anti-phase tACS were tested first. To ensure that there were no systematic differences between in-phase and anti-phase sessions before the application of tACS, permuted two-tailed paired *t*-tests were conducted for Baseline data in Experiment 1. There were no significant differences in accuracy (t(38) = 0.039, *p* = 0.956), reaction time (RT) (t(38) = −0.135, *p* = 0.894), or rate correct score (RCS) (t(38) = −0.002, *p* = 0.998) at Baseline for the 2 stimulation sessions. Further, there were no significant differences between the 2 sessions in Baseline alpha power or frontoparietal alpha synchronization values (alpha power: t(38) = −0.317, *p* = 0.747; frontoparietal alpha synchronization: t(38) = −1.658, *p* = 0.104; permuted paired *t*-tests).

Participants performed the modified Sternberg task with high accuracy and fast reaction times, as is typical of young healthy participants: the mean accuracy across tACS conditions at Baseline is 0.857 (SD = 0.064), and the mean reaction time at Baseline is 878 ms (SD = 134 ms). These behavioral findings in our study are comparable to previous studies using this task [6].

### 2.2. Anti-phase tACS impaired working memory performance compared to in-phase tACS

As many previous brain stimulation studies have noted that stimulation timing modulates stimulation effects (i.e., the inconsistency between the effects during stimulation application and the effects after stimulation application) [28–30], we analyzed the Online tACS effects and Offline tACS effects separately.

**Fig 4:**
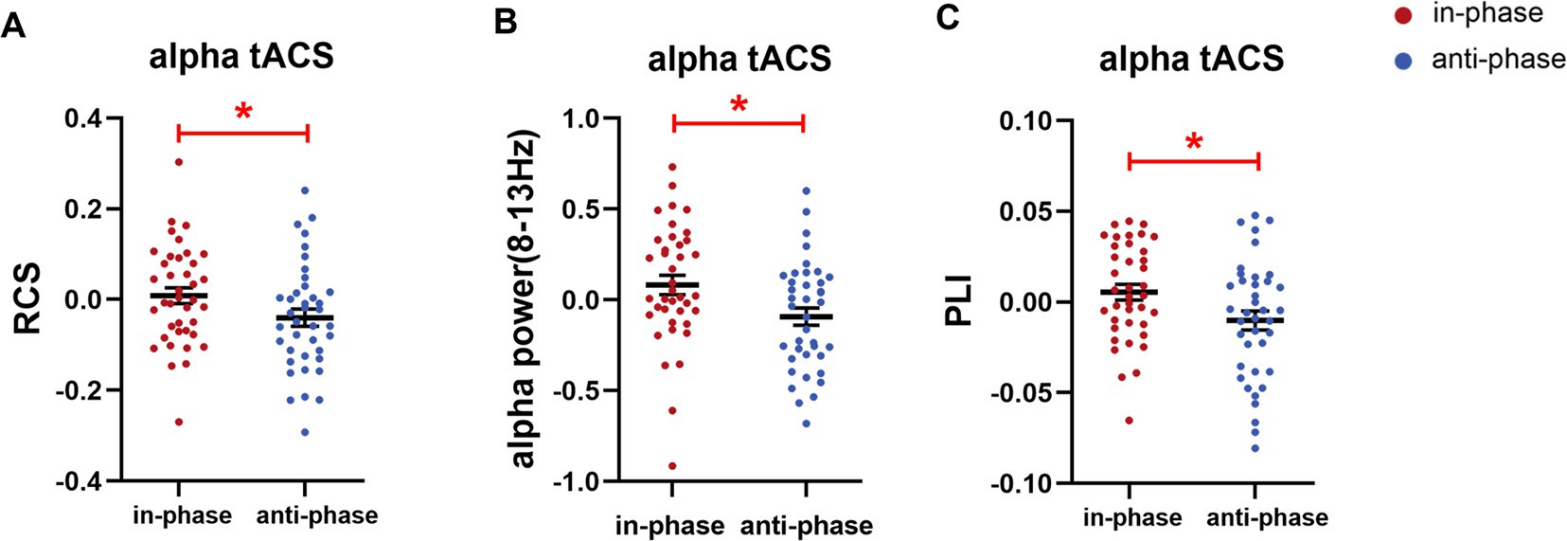
The behavioral and electrophysiological effects of parietal alpha tACS in Experiment 1. During Online tACS, anti-phase tACS at the IAF significantly decreased (**A**) WM performance (i.e., RCS), (**B**) parietal alpha power (8–13 Hz), and (**C**) frontoparietal alpha synchronization (indexed by PLI) as compared to in-phase tACS. Note that all instances of the RCS, alpha power, and PLI values are given relative to the Baseline data (i.e., values after subtracting the corresponding Baseline values). Error bars represent the SEM across participants. *Significant at *p* < 0.05 (two-tailed permuted paired *t*-tests). The underlying data supporting Fig 4 can be found in the Supporting information as S1 Data. IAF, individual alpha frequency; PLI, phase lag index; RCS, rate correct score; SEM, standard error of the mean; tACS, transcranial alternating current stimulation; WM, working memory.

We first investigated whether anti-phase tACS down-regulated WM performance compared to in-phase tACS as hypothesized, measured by RCS, accuracy, and RT. During Online tACS, anti-phase tACS induced a significant RCS reduction compared to in-phase tACS (t(38) = −2.209, *p* = 0.036, Cohen’s d = 0.354; permuted paired *t*-test), which reflected fewer correct responses per second of activity in anti-phase tACS (**Fig 4A**). Anti-phase tACS also induced a marginally significant reduction in accuracy when compared to in-phase tACS (t(38) = −1.958, *p* = 0.057, Cohen’s d = 0.314; permuted paired *t*-test) (**S3A Fig**). No significant difference in RT was observed between in-phase and anti-phase tACS during Online tACS (**S3B Fig**).

### 2.3. Anti-phase tACS suppressed alpha power compared to in-phase tACS

We next assessed whether in-phase tACS and anti-phase tACS modulated the targeted parietal alpha oscillations during stimulation. Pz electrode was chosen for alpha power analysis because its signals served for triggering tACS and it was near to the stimulation electrode. As hypothesized, anti-phase tACS significantly suppressed the alpha power at Pz electrode compared to in-phase tACS during Online tACS (t(38) = −2.329, *p* = 0.023, Cohen’s d = 0.373; permuted paired *t*-test) (**Fig 4B**). This finding suggests the modulation effects of the phase-corrected tACS on the targeted brain activity and the influence of phase differences on tACS electrophysiological effects.

To support the frequency-specific effects of the online phase-corrected tACS, we next tested the influences of alpha tACS on the full physiological frequency band of the EEG (1 to 45 Hz). No significant differences between in-phase tACS and anti-phase tACS were found in any of the frequency bands except for the alpha band during Online tACS (**S4 Fig**). This result indicates the frequency-specific modulation of phase-corrected alpha tACS on endogenous brain oscillations as previous tACS studies have shown [12,31]. Moreover, we also calculated the modulation effects of alpha-tACS on individual alpha power (IAF ± 2 Hz) at Pz electrode and found that anti-phase tACS also showed suppression effects on individual alpha power (**S5 Fig**), further supporting the modulation effects of alpha-tACS on parietal alpha oscillations. Therefore, it is reasonable to attribute the behavioral effects of tACS to the modulation on alpha oscillations at the retention subprocess, supporting the causal link between alpha oscillations and WM retention.

### 2.4. Anti-phase tACS disturbed frontoparietal alpha synchronization compared to in-phase tACS

Previous studies have reported the potential impact of tACS on functional connectivity [32,33] and found increases in the strength of alpha synchronization with increasing memory load among the frontoparietal regions known to underlie executive and attentional functions during WM retention [34,35]. Therefore, Online tACS effects in frontoparietal alpha synchronization were also assessed.

Compared with in-phase tACS, anti-phase tACS induced a significant decrease in frontoparietal alpha synchronization indexed by phase lag index (PLI) during Online tACS (t(38) = −2.067, *p* = 0.044, Cohen’s d = 0.331; permuted paired *t*-test) (**Fig 4C**). Supporting the reliability and reproducibility of these results calculated using PLI, we again detected the anti-phase tACS-induced disturbance when we used another index (weighted PLI) that has been reported to offer an advantage in terms of reduced sensitivity to unrelated noise sources [36] (**S6 Fig)**. These results suggest that the online phase-corrected tACS not only affect the brain activity of the targeted region, but also modulate the connectivity of distributed brain regions.

### 2.5. The correlations between tACS-induced changes in alpha activity and RCS were significantly positive for in-phase condition, which was different from anti-phase condition

**Fig 5:**
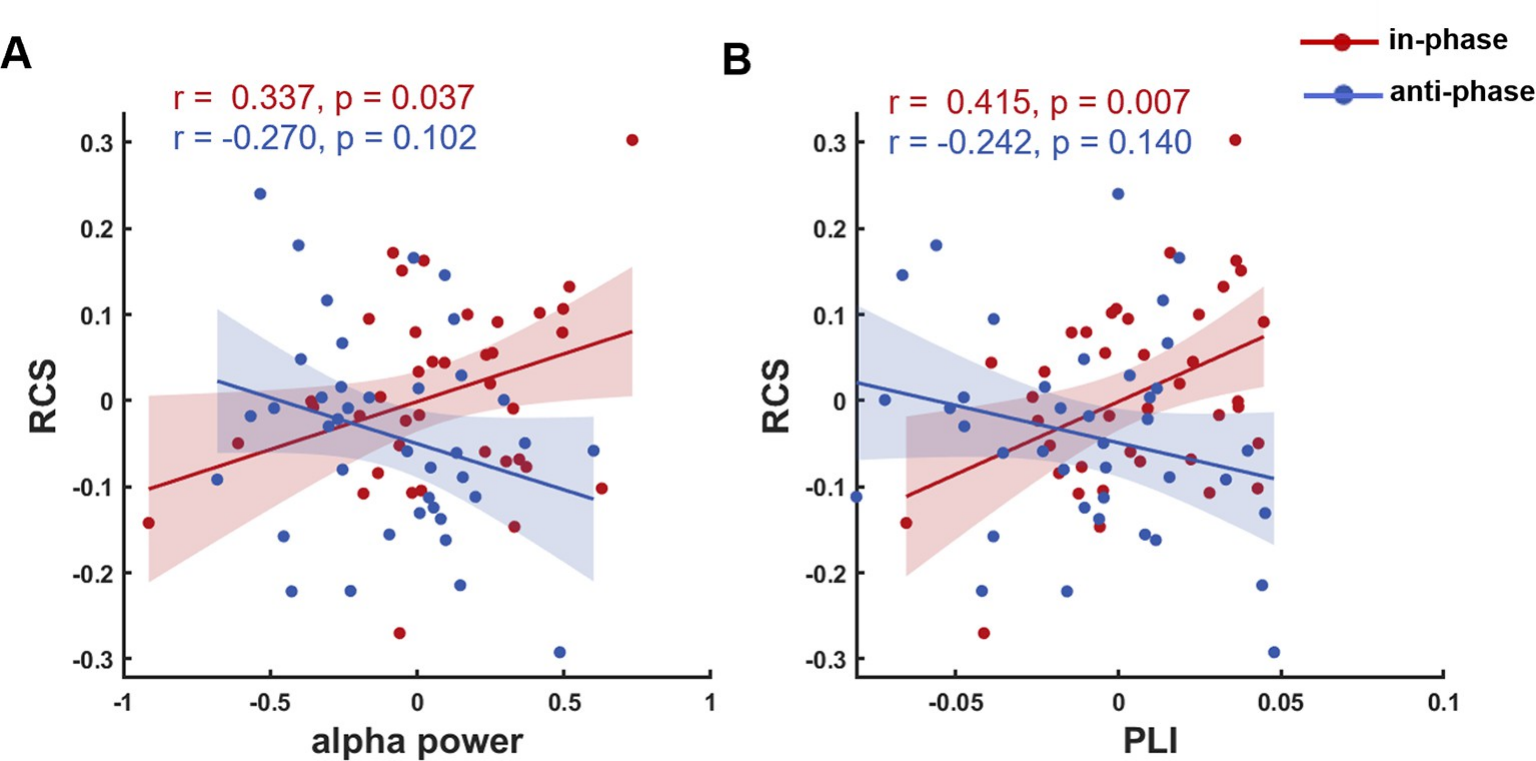
Relationships between tACS-induced changes in RCS and EEG metrics in Experiment 1. The in-phase tACS-induced changes in RCS were positively correlated with (**A**) the changes in alpha power, and (**B**) the changes in PLI; no correlation was found for anti-phase tACS. The RCS, alpha power, and PLI values are given relative to the Baseline values (i.e., after subtracting corresponding Baseline values). The underlying data supporting Fig 5 can be found in the Supporting information as S1 Data. EEG, electroencephalogram; PLI, phase lag index; RCS, rate correct score; tACS, transcranial alternating current stimulation.

As alpha-tACS not only altered RCS, but also modulated alpha power and frontoparietal alpha synchronization during Online tACS, we further investigated whether the modulation of alpha activity related to WM performance. We performed permuted Pearson’ s correlations between the stimulation-induced changes (from Baseline to Online tACS) in RCS and EEG metrics. The correlation between in-phase tACS-induced changes in alpha power and RCS was significant during Online tACS (r = 0.337, *p* = 0.037; permuted Pearson’s correlation). This finding indicated that across participants, increased alpha power related to larger RCS further supporting the causal role for alpha oscillations in WM retention. No correlation was found between anti-phase tACS-induced changes in alpha power and RCS (r = −0.270, *p* = 0.102; permuted Pearson’s correlation), possibly because anti-phase tACS disrupted the inherent relationships between parietal alpha oscillations and WM performance in the absence of exogenous disturbance. Notably, there was a significant difference between the correlation coefficients in in-phase tACS and anti-phase tACS during Online tACS (Z = 2.661, *p* = 0.008) (**Fig 5A**).

Similar to the correlations between the changes in alpha power and RCS, we also found a positive correlation between tACS-induced changes in frontoparietal alpha synchronization and RCS during Online tACS for in-phase tACS (r = 0.415, *p* = 0.007; permuted Pearson’s correlation) and no correlation for anti-phase tACS (r = −0.242, *p* = 0.140; permuted Pearson’s correlation). A significant difference between the correlation coefficients in in-phase tACS and anti-phase tACS was also observed during Online tACS (Z = 2.921, *p* = 0.003) (**Fig 5B**). The observed inverse correlation trends for in-phase tACS and anti-phase tACS further illustrate the different effects between in-phase tACS and anti-phase tACS on WM.

### 2.6. The effects of random-phase tACS and the offline tACS effects

To explore the effects of the control condition in Experiment 1, here we compared the effects of random-phase tACS with both in-phase and anti-phase tACS. During Online tACS, random-phase tACS-induced changes in WM performance (**S7 Fig**), the alpha power at Pz electrode (**S8A Fig**) and frontoparietal alpha synchronization (**S8B Fig**) were all between in-phase tACS and anti-phase tACS. The correlations between random-phase tACS-induced changes in alpha activity and RCS were not significant, also between in-phase tACS and anti-phase tACS (**S9 Fig**). These results indicate the effects of random-phase tACS were between in-phase tACS and anti-phase tACS, further supporting the influence of the phase differences on tACS effects.

Next, we explored the Offline tACS effects (tACS effects beyond the stimulation period: Offline tACS) on WM performance and brain activity. We observed no significant difference between in-phase tACS and anti-phase tACS in WM performance, alpha power at Pz electrode, and frontoparietal alpha synchronization (**S10 Fig**).

### 2.7. The phase-dependent tACS effects were replicated in another sample

In Experiment 2, we sought to replicate the principle findings in a new cohort of participants and examined whether differential cognitive demands influence the efficacy of online phase-corrected tACS, specifically by dividing the trials of the Sternberg task into 2 difficulty levels. Participants were asked to remember 7 consonants in half of the trials (“7-letter trials”) and asked to remember 5 consonants in the other half (“5-letter trials”). The tACS parameters of Experiment 2 were the same as Experiment 1, except that the control condition was changed to sham stimulation (see details in S1 Text: “8. Experiment 2”).

**Fig 6:**
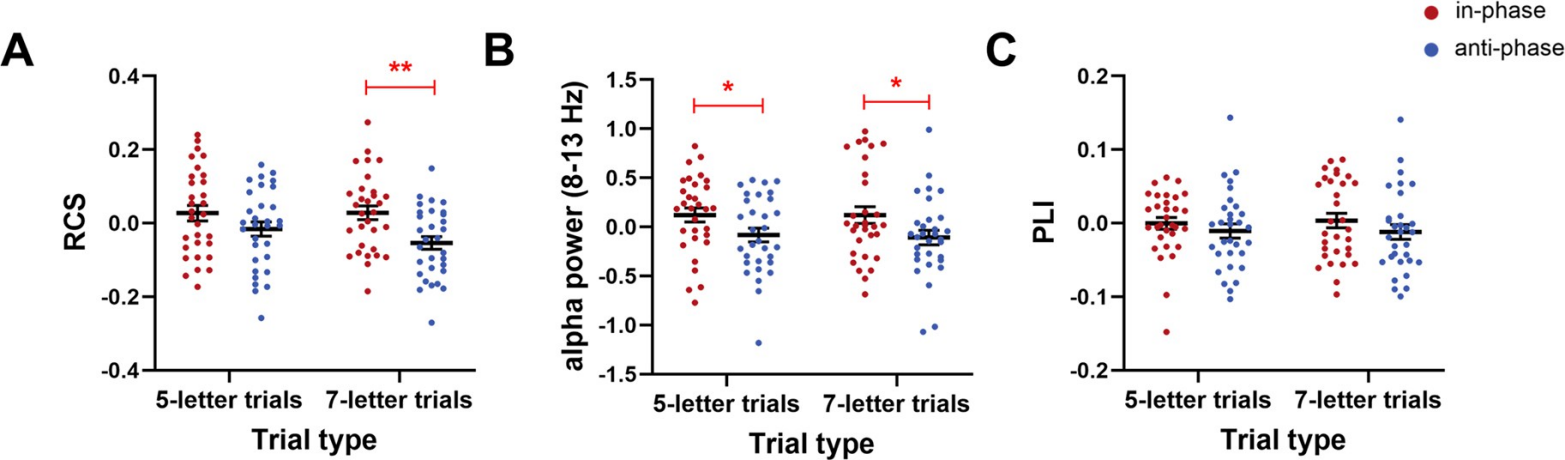
The behavioral and electrophysiological effects of parietal alpha tACS in Experiment 2. **(A)** When compared to in-phase tACS, anti-phase tACS significantly suppressed WM performance (i.e., RCS) in 7-letter trials and showed a tendency to decrease WM performance in 5-letter trials. **(B)** When compared to in-phase tACS, anti-phase tACS significantly suppressed parietal alpha power in either 5-letter trials or 7-letter trials. (**C**) Although not statistically significant, there was a trend for anti-phase tACS to decrease PLI when compared to in-phase tACS in either 5-letter trials or 7-letter trials. Note that all instances of the RCS, alpha power, and PLI values are given relative to the Baseline data (i.e., values after subtracting the corresponding Baseline values). Error bars represent the SEM. *Significant at *p* < 0.05, **significant at *p* < 0.01. The underlying data supporting Fig 6 can be found in the Supporting information as S1 Data. PLI, phase lag index; RCS, rate correct score; SEM, standard error of the mean; tACS, transcranial alternating current stimulation; WM, working memory.

tACS effects were first analyzed using permuted two-way repeated-measures ANOVAs, with trial type (5-letter trials or 7-letter trials) and tACS condition (in-phase tACS or anti-phase tACS) as within-subject factors. During Online tACS, we observed a significant main effect of tACS condition on both RCS and parietal alpha oscillations (RCS: F(1,30) = 9.645, *p* = 0.006, *ɳ*_p_^2^ = 0.243; parietal alpha oscillations: F(1,30) = 5.698, *p* = 0.027, *ɳ*_p_^2^ = 0.160) (**Fig 6A and 6B**), thus replicating the phase-dependent tACS effects. Although not statistically significant, there was a trend for anti-phase tACS to decrease frontoparietal alpha synchronization when compared to in-phase tACS (F(1,30) = 1.928, *p* = 0.171) (**Fig 6C**).

Subsequently, to examine the tACS effects in terms of distinct cognitive demands, the tACS effects in the 5-letter trials and 7-letter trials were investigated separately. For the 7-letter trials, anti-phase tACS significantly decreased both WM performance and parietal alpha oscillations (RCS: t(30) = −3.224, *p* = 0.003, Cohen’s d = 0.579; parietal alpha oscillations: t(30) = −2.062, *p* = 0.048, Cohen’s d = 0.370), consistent with the results from Experiment 1. We also detected a tendency for anti-phase tACS to decrease frontoparietal alpha synchronization when compared with in-phase tACS (t(30) = −1.353, *p* = 0.178). Like 7-letter trials, the anti-phase tACS in the 5-letter trials also significantly decreased parietal alpha oscillations and showed a tendency to decrease WM performance and frontoparietal alpha synchronization as compared to in-phase tACS (RCS: t(30) = −1.504, *p* = 0.145; parietal alpha oscillations: t(30) = −2.303, *p* = 0.027, Cohen’s d = 0.414; frontoparietal alpha synchronization: t(30) = −0.823, *p* = 0.410). We also examined whether the positive brain-behavior correlations in in-phase tACS were replicated. For either 5-letter trials or 7-letter trials, the changes in RCS were not correlated with the changes in parietal alpha power or the changes in PLI (all ps > 0.1).

### 2.8. Frequency-specific effects of tACS on WM performance

**Fig 7:**
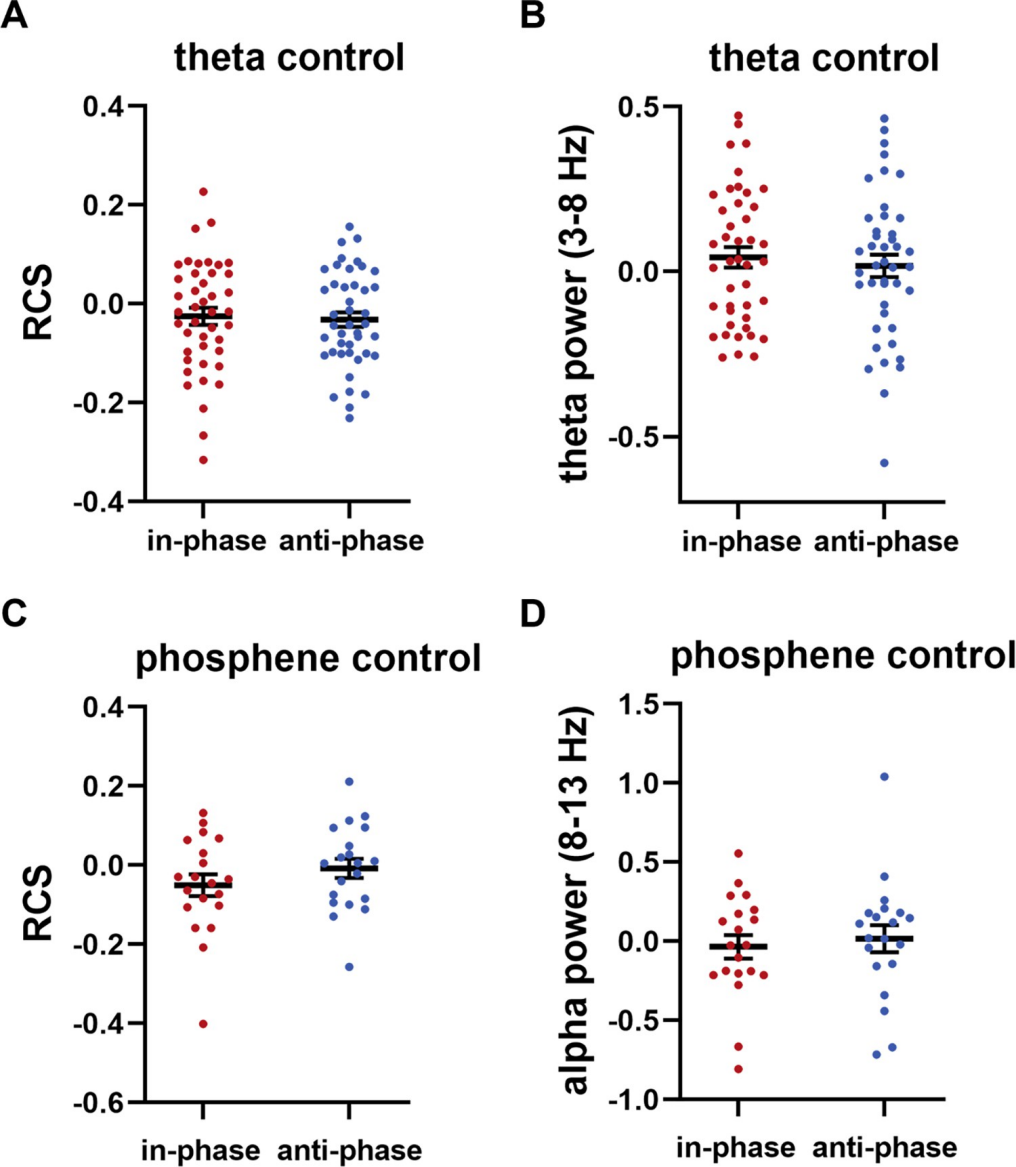
The effects of theta control experiment and phosphene control experiment. The effects of in-phase tACS at the theta frequency band did not differ from the effects of anti-phase tACS for either (**A**) WM performance (i.e., RCS) or (**B**) parietal theta power (3–8 Hz). When tACS was applied at the vertex region, in-phase tACS did not alter (**C**) WM performance or (**D**) parietal alpha power (8–13 Hz) as compared to anti-phase tACS. Note that all instances of the RCS, theta power, and alpha power values are given relative to the Baseline data (i.e., values after subtracting the corresponding Baseline values). Error bars represent the SEM. The underlying data supporting Fig 7 can be found in the Supporting information as S1 Data. RCS, rate correct score; SEM, standard error of the mean; tACS, transcranial alternating current stimulation; WM, working memory.

In Experiment 3, we sought to provide additional support to the conclusion that the observed changes in WM performance can be attributed to changes in parietal alpha oscillations during the WM retention subprocess, rather than to some general effects from tACS (independent of frequency) [27]. Both in-phase tACS (0° relative phase difference to theta oscillations at Pz electrode) and anti-phase tACS (180° relative phase difference to theta oscillations at Pz electrode) were delivered in the theta frequency band (3 to 8 Hz) over parietal-occipital cortex to 43 participants (for more details about theta-tACS experiment, see S1 Text: “10. Experiment 3”). Our choice of theta tACS as a control was motivated by multiple EEG and MEG studies suggesting that local frontal—rather than parietal—theta oscillations are related to WM retention in the Sternberg task [37]. Parietal-occipital theta power during the retention subprocess of the Sternberg task does not increase for the memory condition when compared to the control condition [37] and does not increase with memory difficulty [6,37,38]. The results of Experiment 3 showed no differences between in-phase tACS and anti-phase tACS in WM performance (RCS) (t(42) = 0.313, *p* = 0.757; permuted paired *t*-test) (**Fig 7A**). Moreover, the behavioral effects induced by theta tACS differed from the effects of alpha tACS (see S1 Text: “10.3. The effects of theta tACS differed from alpha tACS”). Parietal theta tACS also failed to modulate targeted theta oscillations: no differences between in-phase tACS and anti-phase tACS were detected for parietal theta power (t(42) = 0.602, *p* = 0.549; permuted paired *t*-test) (**Fig 7B**). This lack of any detected impact from theta-tACS supports that modulation of alpha oscillations—rather than some general impacts from electrical stimulation—can explain the observed effects of alpha-tACS.

### 2.9. tACS effects are unlikely to be caused by retinal stimulation

During tACS, a portion of the current applied to the head travels along the scalp and arrives into the eyes, which has been reported to elicit retinal activation [39,40] and modulatory effects on brain oscillations and memory performance [41,42]. To examine whether the detected phase-dependent tACS effects result from retinal effects rather than cortical effects, we compared participants’ phosphene perception between different tACS conditions and conducted the following phosphene control experiment (Experiment 4).

Participants in Experiment 1 assessed their perception of phosphene immediately after Online tACS. The phosphene perception did not differ significantly between the in-phase tACS and anti-phase tACS (t(38) = 0.658, *p* = 0.515; permuted paired *t*-test), indicating that suprathreshold phosphene perception is unlikely to produce the detected phase-dependent tACS effects. To further examine whether the detected phase-dependent tACS effects could result from retinal effects, we conducted Experiment 4. In Experiment 4, in-phase tACS (0° relative phase difference to alpha oscillations at the Pz electrode) and anti-phase tACS (180° relative phase difference to alpha oscillations at the Pz electrode) were applied over the vertex region. As the vertex region is thought to be irrelevant to WM and has been used as a control site in previous WM-related NIBS studies [43,44], the vertex was chosen to preserve the potential retinal effects in Experiment 1 and exclude the cortical effects (see details in S1 Text: “11. Experiment 4”). We reasoned that if no differential effects were detected between in-phase tACS and anti-phase tACS, the retinal effects could be excluded. The results of Experiment 4 showed that no differences between in-phase tACS and anti-phase tACS were observed in WM performance (t(19) = −1.196, *p* = 0.236; permuted paired *t*-test) or parietal alpha oscillations (t(19) = −0.676, *p* = 0.505; permuted paired *t*-test) (**Fig 7C and 7D**). To show that the effects induced by vertex alpha tACS differed from the effects of parietal alpha tACS (assessed by the data from Experiment 1), we performed additional permuted two-way mixed ANOVAs [45]; 2×2 ANOVAs—stimulation location: vertex and parietal cortex, tACS condition: in-phase tACS and anti-phase tACS—revealed a significant stimulation location × tACS condition interaction for RCS (F(1, 57) = 5.147, *p* = 0.028, *ɳ*_p_^2^ = 0.083) and parietal alpha power (F(1, 57) = 3.464, *p* = 0.036, *ɳ*_p_^2^ = 0.057). Thus, retinal effects could not explain the phase-dependent tACS effects that we observed in Experiment 1.

## 3. Discussion

In this study, we used an online phase-corrected tACS system to modulate alpha oscillations specifically during the WM retention subprocess on a trial-by-trial level, with a predetermined phase difference between the tACS and the concurrent endogenous oscillatory activity. In Experiment 1, compared to in-phase tACS, anti-phase tACS decreased WM performance, and these decreases were paired with corresponding decreases in alpha power and in frontoparietal alpha synchronization at the stimulation period. Notably, the detected changes in alpha power and frontoparietal alpha synchronization induced by in-phase tACS were both positively correlated to behavioral changes. In Experiment 2, the principle behavioral and electrophysiological findings were replicated in a new cohort of participants. Our study therefore provides direct causal evidence of a specific functional impact of alpha oscillatory activity in human WM retention. Our work also illustrates that phase differences between tACS and the targeted brain oscillations represent a decisive factor for determining the effects of tACS, both on neural modulation and on behavioral performances.

### 3.1. Causal role for alpha oscillations in the retention subprocess of WM

Our behavioral and electrophysiological findings strongly support a causal link between parietal alpha oscillations and WM retention. Alpha oscillations during WM retention subprocess have been suggested by previous EEG studies to be instrumental in transiently protecting the encoded memory information by filtering task-irrelevant input and preventing further sensory processing that could interfere with the stored information [5,7,46,47]. To investigate the causal role for alpha oscillations in WM retention, we used the online phase-corrected tACS system to modulate parietal alpha oscillations specifically during WM retention, while also recording EEG signals during intervals without tACS artifacts to investigate the influence of external modulation on alpha oscillations. We found that parietal alpha tACS modulated both WM performance at the stimulation period. Moreover, the behavioral modulation was paired solely with the modulation of brain oscillations in the alpha frequency band (**S4 Fig**). In Experiment 1, we also observed a positive association between in-phase tACS-induced changes in parietal alpha power and the changes in WM performance, further supporting the causal link between alpha activity and WM retention.

A few previous NIBS studies have also attempted to investigate the causal relationship between alpha oscillations and WM in humans [48–50]. Our study is distinct from those studies in at least 2 ways. First, previous studies focused on behavioral effects and did not report a direct impact of NIBS on EEG activity in the alpha frequency band; thus, their causal interpretations remain speculative [48–50]. Our study conducted EEG recordings to support that the modulation of alpha oscillations does represent a plausible physiological basis for the observed behavioral effects. Second, our study applied tACS specifically during the retention subprocesses of WM, rather than continuously throughout the whole process [48]. This enables exclusion of attribution of observed effects to the encoding or the retrieval subprocesses of WM.

### 3.2. Frontoparietal alpha synchronization contributes to WM performance

Our findings also suggest that frontoparietal alpha synchronization contributes to WM. In Experiment 1, anti-phase tACS induced a significant decrease in frontoparietal alpha synchronization at the stimulation period. This decrease may be attributed to disturbance of the parietal alpha phases by anti-phase tACS, which likely impairs its coupling with frontal brain areas. A few studies have found that synchrony is strengthened with increasing memory load in frontoparietal regions previously shown to mediate attentional functions during memory retention [34,35]. In line with these studies, we observed a positive association between in-phase tACS-induced changes in frontoparietal alpha synchronization and altered RCS values, which demonstrates that increased frontoparietal alpha synchronization during WM retention also contributes to the observed improvement in WM performance.

### 3.3. The phase differences between tACS and endogenous brain oscillations affect tACS effects

In agreement with previous computational modeling predictions [21] and animal study [22] suggesting that tACS with different phases relative to endogenous brain oscillations would induce different modulation effects on electrophysiological activities, we experimentally demonstrated that anti-phase tACS significantly inhibited parietal alpha activity and WM performance as compared to in-phase tACS at the stimulation period. To further investigate whether in-phase tACS improve or anti-phase tACS suppress WM performance and alpha activity, we compared the original values measured at Baseline with the original values measured during Online tACS and detected the suppression effects for anti-phase tACS (see more details in S1 Text: “5. Compared to Baseline, anti-phase tACS significantly decreased WM performance and alpha power during Online tACS”). Therefore, we provide the direct experimental evidence that the phase of endogenous brain oscillations relative to tACS is impactful for determining the direction and magnitude of tACS effects.

We speculate that both entrainment and plastic changes may contribute to the detected phase-dependent tACS effects. A previous study examining the mechanisms underlying tACS effects beyond tACS application suggested that these effects could be attributed to plastic changes [51]. Considering that our EEG data were collected during the intermittent, tACS-free intervals—rather than simultaneously during the application of each tACS train—we infer that plastic changes could contribute to the detected phase-dependent tACS effects. Nevertheless, given that tACS-induced online entrainment has been suggested as a window during which effects beyond tACS application can be formed [31], we cannot rule out contributions from online entrainment on the detected phase-dependent tACS effects. To garner further evidence for phase-dependent online entrainment, future investigations could try to apply simultaneous EEG-tACS recordings to explore whether the phase-dependent tACS effects can be detected during tACS application.

The influence of phase differences on tACS effects also calls for the consideration of brain states during tACS (e.g., the phase differences between the tACS waveform and the endogenous alpha oscillations) to overcome the unwelcome inconsistent effects of conventional open-loop tACS [16]. The suppression effects of anti-phase tACS compared to in-phase tACS also suggest that tACS can act by the modulation of cortical neurons, rather than through peripheral nerve stimulation in the scalp [52]. Effects from in-phase tACS versus anti-phase tACS should not differ if they only result from transcutaneous stimulation [52].

### 3.4. The reproducibility of phase-dependent tACS effects

The reproducibility of the phase-dependent tACS effects in a new cohort of participants boosts the effect stability and confidence of the observed patterns. In the 7-letter trials of Experiment 2, which were consistent with Experiment 1 in terms of experimental designs, anti-phase tACS significantly decreased WM performance and parietal alpha power as compared to in-phase tACS. These effects were consistent with the results from Experiment 1. When we further combined the data from Experiment 1 and the data from the 7-letter trials of Experiment 2 to enlarge sample size, anti-phase tACS significantly decreased WM performance, parietal alpha power, and frontoparietal alpha synchronization (**S13 Fig**). These results lend further support to the core findings of our research: (1) parietal alpha oscillations are causally linked to WM retention; (2) the phase differences between tACS and brain oscillations influence the direction and magnitude of tACS effects.

Some results of Experiment 1 were not replicated in Experiment 2, which might result from the confounding effects in experimental design. The lower cognitive demand in the 5-letter trials (**S11 Fig**) may result in the less involvement of parietal alpha oscillations [6,11] and frontoparietal network [53], thus resulting in the absence of significant tACS effects on WM performance and frontoparietal alpha synchronization in the 5-letter trials [54,55]. As 5-letter trials and 7-letter trials were randomly presented and participants reported switching memory strategies between the 2 types of trials, Experiment 2 may engage cognitive flexibility. The negative relationship between alpha activities and cognitive flexibility [56,57] may interfere with the positive brain-behavior correlations detected in Experiment 1, resulting in the absence of brain-behavior correlation in in-phase tACS. Moreover, the reduced number of trials in Experiment 2 may also prevent the detection of significant effects [58]. To better replicate the results of Experiment 1, future investigations could try to conduct the 5-letter trials and 7-letter trials in 2 separate studies.

### 3.5. The advantages and application potentials of the online phase-corrected tACS

The capacity to adjust the direction of tACS effects on brain oscillations within a relatively short period of time (within several seconds) is a unique and highly valuable feature of our online phase-corrected tACS system as compared to other currently available tACS techniques. Conventional continuous tACS was usually demonstrated to enhance brain oscillations at the stimulation frequency via entrainment [13]. Although several studies reported the suppression effects of conventional long-term continuous tACS on brain oscillations at the non-stimulation frequency during specific brain states [59,60] (e.g., the suppression effects of alpha tACS on gamma oscillations in a visual detection task [60]), this kind of inhibition effects cannot be generalized because the correspondence between the tACS frequency and the frequencies of the suppressed brain oscillations is not clear. Moreover, recently published brain state-dependent tACS set-ups have to date only reported unidirectional modulation of brain oscillations at the stimulation frequency [61–64]. This inflexibility regarding directionality of tACS effects within a short period of time is problematic for many functions and applications. Consider for example that previous studies of the Sternberg paradigm have indicated that alpha activity tends to increase during the retention subprocess but to decrease during the encoding and retrieval subprocesses [24,25]. If conventional long-term (several to dozens of minutes) continuous tACS was performed throughout the entire task process, the effects in different subprocesses might cancel each other out. We selectively regulated alpha oscillations during the retention subprocess; future studies could try to up-regulate alpha activity during the retention subprocess while also—and in the same trial—down-regulating oscillatory alpha activity or even manipulating related oscillations at other frequencies during the encoding and retrieval subprocesses. This method, or many variations thereof, may prove to be an even more effective way of modulating cognitive processes, e.g., working memory.

It is further plausible to speculate that our online phase-corrected tACS system may broaden the clinical therapy applications of tACS, with its expanding capacity to suppress brain oscillations within a relatively short period. Our results show that anti-phase tACS can significantly inhibit oscillatory activity, illustrating the possibility of using tACS to down-regulate abnormally high brain rhythms, especially those excessive brain oscillations that only occur within a short time during the attacks of diseases. For example, our anti-phase tACS may be suitable to suppress the cue-induced excessive low-frequency oscillations (e.g., increased delta or theta oscillations) of cocaine addicts within a short period [65], which cannot be achieved by conventional open-loop tACS without considering the phase differences.

### 3.6. Limitations

The present study has several limitations. First, we did not adjust the tACS frequency to match the real-time alpha peak frequency in a trial-by-trial manner, owing to the low signal-to-noise ratio of the single-trial EEG data, which could restrict the precise phase alignment between tACS and alpha oscillations. Perhaps future studies could explore adjusting the stimulation frequency in a trial-by-trial manner to realize more precise phase alignment. Second, there was no difference in behavioral performance between the random-phase tACS and anti-phase tACS (**S7 Fig**), but the difference in parietal alpha power was observed between the 2 tACS conditions (**S8 Fig**). The mismatch in parietal alpha power and behavioral performance could be interpreted to suggest that noise could have somehow influenced the measurements of random-phase tACS. To explore an alternative control condition, we conducted Experiment 2, which employed a sham stimulation as the control. We found no evidence for the mismatch in the sham condition (**S12 Fig**), while participants could distinguish between sham condition and the real stimulation conditions (see the S1 Text: “8.5. Sham stimulation caused the problem of blind breaking”). In the intermittent phase-corrected tACS protocols, the appropriate control condition needs further exploration.

### 3.7. Conclusions

In summary, our study provides empirical evidence for a causal link between parietal alpha oscillations and the retention subprocess of WM. To establish this link, we here pioneered an online phase-corrected tACS system that offers the possibility of directly up- and down-regulating oscillatory brain activities at a predetermined stimulation frequency, and we show that this system does induce effects on both brain rhythms and behavior.

## 4. Materials and methods

### 4.1. Participants

We calculated the needed sample size using G*Power 3.1 software [66]. Experiment 1 investigated whether anti-phase alpha-tACS and in-phase alpha-tACS would induce different behavioral and electrophysiological effects. Based on the medium or large effect sizes reported in previous studies that used NIBS to modulate parietal alpha oscillations and WM [48,50], a medium effect size (Cohen’s d = 0.5) was assumed. Thus, a sample size of 34 participants was needed to detect a reliable effect with a statistical power of 80% and an alpha probability of 0.05 in a two-tailed paired *t*-test.

A total of 48 healthy participants between the ages of 18 and 40 completed Experiment 1. All participants were right-handed and reported no metal implants in the brain, no implanted electronic devices, no history of neurological problems or head injury, no current use of psychoactive medication, no history of craniotomy, and no skin sensitivity; the participants were nonpregnant, had normal or corrected-to-normal visual acuity, and were not enrolled in any other NIBS research within 3 months of their study participation. Participants who failed to follow directions or did not understand instructions were removed from the study. All participants were recruited in Hefei, China through advertisements or posters. Eight participants were excluded from the analyses due to poor EEG signals or malfunctions of the tACS stimulator to trigger the intended electrical stimulation. One participant whose alpha activity was too weak for detection and who therefore had to repeat the Baseline Sternberg task 3 times per session was also excluded. The remaining 39 participants (19 females, mean age ± SD:21.1 ± 2.2 years, mean education ± SD:14.7 ± 1.8 years) were included for behavioral and EEG analyses, which meets the sample size requirement.

### 4.2. Ethics statement

The study was approved by the Human Ethics Committee of the University of Science and Technology of China (IRB No.: 2020KY161) and performed in accordance with the latest Declaration of Helsinki. All participants provided written informed consent prior to the study.

### 4.3. Experimental procedures

In Experiment 1, participants attended 3 sessions, 1 for each of the 3 distinct tACS conditions (in-phase tACS, random-phase tACS, or anti-phase tACS) were applied (**Fig 3D**). The order of tACS conditions was counterbalanced across participants (**Fig 3B**). The 3 sessions of each participant were all separated by at least 3 days and performed at approximately the same time of the day. The experimental procedures for each session are as follows (**Fig 3C**).

At the start of each session, participants first completed 32 practice trials of the Sternberg task (**Fig 3A**). Following this practice, participants performed a total of 300 trials of the Sternberg task divided into 5 sets.

In the first 60 trials (“Baseline”), spontaneous EEG signals were recorded for 2.5 s in each trial, starting from the beginning of a retention subprocess. Following the Baseline trials, the IAF and the threshold were calculated from the Baseline EEG data at the Pz electrode (see more details in S1 Text: “1.2. IAF and threshold determination”). The IAF was used as the subsequent tACS frequency for this session. The threshold was set to restrict the application of tACS to when parietal alpha activity is prevailing to be detected.

Then, participants performed 180 tACS-EEG trials (“Online tACS”) while performing the Sternberg task. In each trial, EEG data at the Pz electrode was monitored continuously once the retention subprocess began. The online phase-corrected tACS was triggered when the amplitude of endogenous alpha oscillations exceeded the threshold twice in a row (see more details in S1 Text: “1.1. The details of online phase-corrected tACS system”). Each tACS application lasted for 0.8 s. Once stimulation was triggered, the EEG recordings stopped to avoid tACS artifacts. When the retention subprocess of the next trial began, EEG recordings restarted and the aforementioned process was again used to determine whether and when tACS was triggered. After Online tACS, participants filled out a tACS sensation questionnaire to assess tACS-induced discomfort (see more details in Blinding section).

Following Online tACS, participants completed the final 60 trials of the Sternberg task, while EEG data were recorded during the retention subprocesses (“Offline tACS”).

### 4.4. Sternberg task

Participants performed a previously published modified version of the Sternberg WM task [6,8] (**Fig 3A**). In Experiment 1, at the beginning of each trial, a black fixation cross was displayed on a gray background for 1 s. Subsequently, participants had to remember a sequence of 7 black consonants that was presented simultaneously at the center of a gray screen for 2 s. After the 7 consonants disappeared, a retention subprocess started and lasted for 3 s, where a black fixation cross was presented at the center of a gray screen. Following the retention subprocess, a probe stimulus was displayed for 1 s. Participants had to decide whether the probe stimulus had been in the sequence just presented by pressing the left arrow with the index finger or the right arrow with the middle finger of the right hand. The probe stimulus was present in the memory sequence at 50% probability. Participants were instructed to respond as quickly and as accurately as possibly. After the probe stimulus disappeared, an inter-trial-interval (1.5 to 2.5 s, uniform random distribution) started, where a fixation cross was presented on a gray background. During the inter-trial-interval, the fixation cross was in black before a button press was detected and turned green to indicate that participants were allowed to blink their eyes once a button press was detected.

### 4.5. EEG data collection

EEG was recorded with Ag/AgCl electrodes at 13 locations according to the international 10 to 20 system [67] (Fp1, Fp2, F7, F3, Fz, F4, F8, C3, Cz, C4, P3, Pz, P4) using an UEA-16BZ amplifier (SYMTOP, Beijing, China). Two additional electrodes were placed over left and right mastoids. As previous studies reporting the relationship between alpha oscillations and WM retention have used mastoids as the reference [6,25]; in our study, EEG was referenced to the average of the mastoid electrodes. The ground electrode was attached to the AFz. Impedances of all electrodes were kept below 5 kΩ. All signals were sampled at 1,000 Hz during data collection. A low-pass filter with a cutoff frequency of 45 Hz and a 50 Hz notch filter were applied.

For the Baseline and Offline trials, EEG recording started from the onset of the retention subprocess and lasted for 2.5 s in each trial. For trials during Online tACS, EEG recordings started from the onset of the WM retention subprocess. Note that the online phase-corrected tACS system kept running for a duration of 1.8 s starting from the onset of a retention subprocess; this allowed us to determine (1) whether and (2) when to apply a short tACS-train in a given trial. Once stimulation was triggered, the EEG recordings were stopped until the onset of the next trial to preclude the acquisition of potential tACS artifacts (**Fig 3A**). For trials where tACS was not triggered, the EEG recordings lasted for 2.5 s, starting from the beginning of a retention subprocess (**Fig 3A**).

### 4.6. Online phase-corrected transcranial alternating current stimulation (tACS)

To specifically stimulate the retention subprocess in each trial with a predetermined phase difference between the applied stimulation and the endogenous oscillatory activities at the target brain region, we developed an online phase-corrected tACS system in the course of this study (**Fig 1**). Brain oscillations were first measured with an EEG module, after which the raw EEG data were analyzed in real-time by a computation module to extract the amplitudes and phases of an underlying brain rhythm. Once the characteristics of the brain oscillations satisfied the predetermined requirements (see S1 Text: “1.1. The details of online phase-corrected tACS system”), the tACS stimulator was triggered to modulate the underlying oscillations. For more details about how to realize the certain phase differences between tACS and brain oscillations, see the S1 Text: “1.1. The details of online phase-corrected tACS system.”

In Experiment 1, tACS was applied at the IAF of each participant, with a peak-to-peak amplitude of 2 mA. tACS was delivered with 3 distinct phase differences relative to endogenous alpha oscillations at the Pz electrode (in-phase tACS, anti-phase tACS, or random-phase tACS) (**Fig 3D**). The endogenous alpha oscillations within the frequency range of IAF ± 2 Hz were extracted from the raw EEG signals in real-time (using a 200th order FIR filter). The frequency range for alpha oscillations (i.e., IAF ± 2 Hz) that we selected was consistent with previous alpha-tACS studies [31,68,69]. The selection of Pz electrode was based on previous studies in which a Pz electrode was reported to exhibit the strongest alpha power among all electrodes during the retention subprocess of the Sternberg task [6]. A stimulation electrode (4 × 6 cm, rubber electrode) was placed over central parietal-occipital cortex (between Pz and Oz, according to the international 10 to 20 system), with the upper edge about 2 cm away and the center about 4 cm away from the Pz electrode. The stimulation electrode was not placed precisely over Pz electrode, given that the rubber stimulation electrode would interfere with EEG recordings at the location where it was placed. The position of the stimulation electrode in our study is suitable due to the high coherencies of EEG signals at about 5-cm electrode separations [70] and the relatively large sources accounting for alpha activity in WM, extending from parietal region to occipital region [8] (S1 Text: “12.1. A discussion regarding positioning of the stimulation electrode”). The return electrode (6 × 9 cm, rubber electrode) was placed over the right shoulder. Impedances of the 2 electrodes were kept below 10 kΩ using Ten20 conductive paste (Nuprep, Weaver and Company, Aurora, Colorado, United States of America), which also held the electrodes in place.

The phase-corrected tACS system ran for about 30 min during Online tACS. To explore the potential roles of alpha oscillations specifically in the retention subprocess, monitoring of alpha oscillations and the application of tACS trains were restricted to the retention subprocess of each trial (i.e., rather than continuously performed during the whole process of each trial, as has been performed in previous WM-related tACS studies [48,71]). To avoid the 0.8 s stimulation beyond the retention subprocess, the monitoring of alpha oscillations for triggering electrical stimulation would stop at the 1.8 s starting from the onset of the retention subprocess. During the monitoring of alpha oscillations, the amplitudes and phases of alpha oscillations at Pz electrode in a moving 500 ms time window (with 10-ms step) were extracted in real-time by the computation module. When the amplitude of endogenous alpha oscillations exceeded the threshold twice in a row, the online phase-corrected tACS was triggered (see more details in S1 Text: “1.1. The details of online phase-corrected tACS system”). Once tACS was triggered, EEG recordings stopped to preclude the acquisition of the potential tACS artifacts and the appropriate time point to initiate the stimulation was transmitted to the tACS stimulator. The tACS stimulator was set to provide a subsequent sinusoidal current stimulation at the IAF lasting for 0.8 s. The onset phases of the 0.8 s sinusoidal waveforms were invariably 0 in all conditions. There were no ramp up and ramp down periods in the 0.8 s stimulation. If tACS was not triggered within 1.8 s, the monitoring of alpha oscillations would stop and the EEG recordings continued to the 2.5 s starting from the onset of the retention subprocess. To ensure the accurate phase alignment between alpha oscillations and tACS waveforms, any time delays associated with data processing or transmission were measured and corrected (see more details in S1 Text: “1.1.2. Delay correction”).

### 4.7. Blinding

Before Online tACS, all participants were exposed to tACS with the same stimulation parameters as Online tACS for no more than 5 times to make sure that the stimulation was acceptable for participants. Only 1 participant reported relatively severe painful sensations and was terminated from the experiment. All participants who completed the experiment confirmed that the stimulation was acceptable during tACS and did not induce severe discomfort after the experiment.

After Online tACS, participants completed a sensation questionnaire to assess 10 possible tACS side effects by rating from 0 (none) to 4 (strong) for the intensity of: itching, pain, burning, warmth/heat, pinching, metallic/iron taste, fatigue, dizziness, nausea, phosphenes, or any other side effects perceived. tACS-induced discomfort was computed as the summation of the intensity score recorded for each single sensation. In Experiment 1, tACS-induced discomfort did not differ between any 2 tACS conditions (in-phase versus anti-phase: t(38) = 0.956, *p* = 0.370; in-phase versus random-phase: t(38) = −0.259, *p* = 0.820; anti-phase versus random-phase: t(38) = −1.212, *p* = 0.249). The lack of differences in stimulation-induced side effects between tACS conditions rule out the possibility that the reported phase-dependent tACS effects resulted from the differences in tACS sensations. These results also indicated that participants cannot distinguish between different tACS conditions and were thus blinded to the tACS condition.

### 4.8. EEG analysis

Analysis of the EEG data was performed using custom-built scripts implemented in MATLAB 2016a (MathWorks, Natick/USA) using the EEGLAB Toolbox (version 13.5.4b) [72].

#### 4.8.1. Preprocessing

The EEG data obtained during Online tACS were epoched first. As the time to trigger electrical stimulation in the retention subprocess was different across trials, the lengths of epochs for different trials were also different. The epochs of the trials with wrong responses in the Sternberg task were deleted. The detrending was then performed to remove DC offsets and slow drifts (<1 Hz). The ocular artifacts were then eliminated using an independent component analysis approach (implemented in EEGLAB) [72]. Epochs with residual ocular or other artifacts were removed through visual inspection of the data. As the epoch numbers and the length of epochs obtained from the 3 tACS conditions were quite different, it was necessary to balance the epoch numbers and epoch lengths of the 3 tACS conditions. Thus, for each participant, we deleted the shortest epochs in the 2 tACS conditions with more epochs so that the epoch numbers were the same as the tACS condition with the minimum number of epochs. Next, the epochs in each tACS condition were sorted by the epoch length in an ascending order. For the 3 epochs with the same order in epoch length among the 3 tACS conditions, we discarded the tail EEG data of the 2 longer epochs to make the epoch length the same across the 3 tACS conditions. For example, we assume that the epoch length of the longest epoch for the in-phase tACS, anti-phase tACS, and random-phase tACS was 1.8 s, 1.6 s, and 1.5 s, respectively. The 0.2 s EEG data at the end of the in-phase epoch and the 0.1 s EEG data at the end of the anti-phase epoch would be deleted.

The Baseline and Offline EEG data were also epoched first, and the epoch length for all Baseline and Offline epochs was 2.5 s. We matched the Baseline and Offline epoch lengths with those during Online tACS. For every participant and every condition, the epoch lengths during Online tACS were randomly assigned to the epochs at Baseline and during Online tACS. To make the epoch length consistent with the assigned length, the EEG data at the end of each epoch was deleted according to the assigned length. For example, assuming that the assigned length of a Baseline epoch was 1.5 s, the 1 s EEG data at the end of the epoch would be deleted. Then, the preprocessing for Baseline and Offline EEG data was the same as the aforementioned preprocessing for the Online EEG data.

After preprocessing, the mean and standard deviation of the number of trials across participants for Baseline, Online tACS, and Offline tACS were 47.51 ± 3.77 (range, 42 to 54), 141.97 ± 11.60 (range, 110 to 165), and 47.59 ± 5.39 (range, 39 to 56) respectively.

#### 4.8.2. Analysis of alpha power (8 to 13 Hz)

For each epoch, the absolute spectrum was calculated using the Matlab function *pwelch*. Each epoch was first divided into several small segments of 500 ms, with an overlap percentage of 50% (i.e., 250 ms) from segment to segment [73,74].

A fast Fourier transform (FFT) was calculated for each segment using a Hamming window and zero-padding to 2.048 s. Then, the spectra of the segments for every epoch were averaged to obtain the spectrum of the epoch. For every condition and every time period (i.e., Baseline, Online tACS, or Offline tACS), the spectra of epochs were averaged across epochs as well as across the alpha-bands (8 to 13Hz) as the absolute power in alpha band [75]. Finally, for subsequent statistical analysis, the relative alpha power was calculated as the absolute power in the alpha band divided by the absolute power across the frequency band of 1 to 45 Hz.

#### 4.8.3. Phase synchronization analysis

The PLI is defined as

$$PLI=|\frac{1}{N}\sum_{n=1}^{N}sign(\Delta (\varphi_{t_{n}}))|$$ (1)

Where sign represents the signum function that gives the sign for the given values of phase difference: for phase difference in the interval (-π, 0), the output is +1; for phase difference in the interval (0, π), the output is −1; for phase difference equal to 0 or π, the output is 0. $\Delta (\varphi_{t_{n}}),n=1\ldots N$ represents a time series of phase differences. As PLI can obtain reliable estimates of phase synchronization against the presence of volume conduction [76], phase synchronization between the Pz electrode and the other 12 electrodes was estimated using the PLI method.

To obtain the phases of alpha oscillations, preprocessed EEG data were bandpass-filtered in alpha-bands (8 to 13 Hz) and the phases of filtered EEG data were obtained via Hilbert transformation. The PLI between Pz electrode and one of the other 12 electrodes was then computed for every epoch. For every condition and every time period (i.e., Baseline, Online tACS, or Offline tACS), PLIs were averaged across epochs. Finally, the averaged PLIs between Pz electrode and the 7 frontal electrodes (Fp1, Fp2, F7, F3, Fz, F4, and F8) were used as a measurement of the frontoparietal alpha synchronization.

### 4.9. Statistical analysis

The RCS is defined as

$$RCS=\frac{c}{\sum RT}.$$ (2)

Where c is the number of correct responses, and the denominator refers to the sum of all RTs (including both correct and incorrect RTs) in the set of trials under consideration.

A previous study proposed that RCS is suitable for assessment of a memory task for integrating measurements of RT and accuracy into a single measure [77]. It can be interpreted directly as the number of correct responses per unit time. Follow-up researches support the utility of RCS in cognitive science [78,79]. This measure is more sensitive and less variable than single measures of RT and accuracy, especially under conditions in which an external manipulation or intervention could cause parallel, rather than reciprocal, changes in reaction time and accuracy (i.e., both disturb or both improve) [80].

As many previous brain stimulation studies have noted that stimulation timing modulates stimulation effects (i.e., the inconsistency between the effects during stimulation application and the effects after stimulation application) [28–30], we analyzed the Online tACS effects and Offline tACS effects separately. To control for the Baseline differences and test tACS-induced behavioral and electrophysiological changes, difference scores were computed for all metrics (including RCS, accuracy, RTs, alpha power, and PLI) by subtracting the corresponding Baseline values from the values measured during Online tACS or Offline tACS.

We used nonparametric permutation tests throughout the study (to avoid the assumption of normality). For comparisons between the 2 paired groups, we used the permuted paired *t*-test statistic wherein we randomly mixed values from the 2 groups 5,000 times to create a distribution of paired *t* values and computed an empirical *p* value from this distribution. For comparisons between more than 2 groups, we performed permutation-based N-way ANOVAs using the teg_RMA toolbox [45]. Note that *t*-test statistics, *F*-statistics, and the degree of freedom were provided for reference only, and the reported *p* values may differ from those expected from the *t*- and *F*-distribution. The effect size (Cohen’s d-value) for permuted paired *t*-tests was calculated via G*Power 3.1 software [66], and the effect size (partial eta squared, *ɳ*_*p*_^*2*^) for permuted N-way ANOVAs was calculated using the teg_RMA toolbox [45]. Correlation analysis between 2 variables was calculated using permutation tests based on Pearson’s linear correlation coefficient (r-value, two-tailed). We used the permuted Pearson’s correlation coefficients wherein we hold 1 variable constant and randomly permuted the other variable 5,000 times to create a distribution of r values and computed an empirical *p* value from this distribution. To test for differences between 2 correlations, the obtained correlation coefficients were converted into z-values with Fisher’s r-to-z transformation. Paired *t* tests were then used to test for differences between correlations.

## Supporting information

S1 Fig The correlation between the system delays of the tACS system and the cycle time of tACS waveform. The system delays were positively correlated with the cycle time of the tACS waveform. The underlying data supporting S1 Fig can be found in the Supporting information as S1 Data. tACS, transcranial alternating current stimulation.
(TIF)

S2 Fig Distribution of the phase differences between EEG signals and tACS waveforms. (**A**) Within the first 0.1 **s,** the mean and the standard deviation of the phase differences between EEG signals and tACS waveforms was 7.36° ± 15.09° for in-phase tACS and 177.52° ± 15.45° for anti-phase tACS. The phase differences are binned (width, 22.5°) and frequencies are indicated (inner ring = 20%, middle ring = 40%, and outer ring = 60%). (**B**) During the whole 0.8 s stimulation across all subjects, the mean and the standard deviation of the phase differences between EEG signals and tACS waveforms was 2.42° ± 52.73° for in-phase tACS and 185.65° ± 44.91° for anti-phase tACS. The phase differences are binned (width, 30°) and frequencies are indicated (inner ring = 20%, outer ring = 40%). The underlying data supporting S2 Fig can be found in the Supporting information as S1 Data. EEG, Electroencephalogram; tACS, transcranial alternating current stimulation.
(TIF)

S3 Fig **The Online effects of tACS on** (**A**) accuracy and (**B**) reaction time for the 2 stimulation conditions: in-phase and anti-phase tACS. Accuracy and reaction time are given relative to Baseline. Error bars represent SEM; + marginally significant at 0.05 < *p* < 0.1. The underlying data supporting S3 Fig can be found in the Supporting information as S1 Data. SEM, standard error of the mean; tACS, transcranial alternating current stimulation.
(TIF)

S4 Fig In Experiment 1, the comparison of power in delta, theta, alpha, beta, and gamma bands between in-phase and anti-phase tACS during Online tACS. Powers are given relative to the corresponding values at Baseline. Error bars represent the SEM; *significant at *p* < 0.05, **significant at *p* < 0.01. The underlying data supporting S4 Fig can be found in the Supporting information as S1 Data. SEM, standard error of the mean; tACS, transcranial alternating current stimulation.
(TIF)

S5 Fig In Experiment 1, the Online effects of alpha-tACS on individual alpha power for the 2 stimulation conditions: in-phase and anti-phase tACS. Individual alpha power is given relative to Baseline. Error bars represent SEM; + marginally significant at 0.05 < *p* < 0.1. The underlying data supporting S5 Fig can be found in the Supporting information as S1 Data. SEM, standard error of the mean; tACS, transcranial alternating current stimulation.
(TIF)

S6 Fig In Experiment 1, the Online effects of tACS on frontoparietal alpha synchronization, indexed by WPLI for the 2 stimulation conditions: in-phase and anti-phase tACS. WPLI is given relative to Baseline. Error bars represent SEM; + marginally significant at 0.05 < *p* < 0.1. The underlying data supporting S6 Fig can be found in the Supporting information as S1 Data. SEM, standard error of the mean; tACS, transcranial alternating current stimulation; WPLI, weighted phase lag index.
(TIF)

S7 Fig The effects of tACS on working memory performance. The change for (**A**) RCS, (**B**) accuracy, and (**C**) reaction time during Online tACS and Offline tACS for the 3 stimulation conditions: in-phase, random-phase, and anti-phase tACS. RCS, accuracy, and reaction time are given relative to Baseline (subtract corresponding Baseline values). Error bars represent SEM. The underlying data supporting S7 Fig can be found in the Supporting information as S1 Data. RCS, rate correct score; SEM, standard error of the mean; tACS, transcranial alternating current stimulation.
(TIF)

S8 Fig EEG results elicited by in-phase, random-phase, and anti-phase tACS conditions. (**A**) The alpha power of Pz electrode in in-phase and anti-phase tACS compared with random-phase tACS. (**B**) The frontoparietal alpha synchronization, indexed by PLI in in-phase and anti-phase tACS compared with random-phase tACS. Alpha power and frontoparietal alpha synchronization are given relative to Baseline (subtract corresponding Baseline values). Error bars represent SEM; + marginally significant at 0.05 < *p* < 0.1, *significant at *p* < 0.05. The underlying data supporting S8 Fig can be found in the Supporting information as S1 Data. EEG, electroencephalogram; PLI, phase lag index; SEM, standard error of the mean; tACS, transcranial alternating current stimulation.
(TIF)

S9 Fig Relationship between tACS-induced changes in RCS and EEG metrics for in-phase, random-phase, and anti-phase tACS. (**A**) Correlation between the changes in RCS and parietal alpha power relative to Baseline at each time point (Online tACS and Offline tACS). (**B**) Correlation between the changes in RCS and frontoparietal alpha synchronization at each time point (Online tACS and Offline tACS). RCS, alpha power, and frontoparietal alpha synchronization are given relative to Baseline (subtract corresponding Baseline values). The underlying data supporting S9 Fig can be found in the Supporting information as S1 Data. RCS, rate correct score; SEM, standard error of the mean; tACS, transcranial alternating current stimulation.
(TIF)

S10 Fig **The Offline effects induced by in-phase tACS and anti-phase tACS in** (**A**) RCS, (**B**) accuracy, (**C**) RT, (**D**) alpha power, and (**E**) frontoparietal alpha synchronization. RCS, accuracy, reaction time, alpha power, and frontoparietal alpha synchronization are given relative to Baseline (subtract corresponding Baseline values). Error bars represent SEM. The underlying data supporting S10 Fig can be found in the Supporting information as S1 Data. RCS, rate correct score; RT, reaction time; SEM, standard error of the mean; transcranial alternating current stimulation.
(TIF)

S11 Fig The Baseline RCS values in the 7-letter trials were significantly lower compared to the 5-letter trials. Note that RCS values are original values. Error bars represent the SEM; ***significant at *p* < 0.001 (two-tailed permuted paired *t*-tests). The underlying data supporting S11 Fig can be found in the Supporting information as S1 Data. RCS, rate correct score; SEM, standard error of the mean.
(TIF)

S12 Fig The effects of sham condition. The effects of sham condition were all intermediate between the in-phase tACS and anti-phase tACS for (**A**) WM performance, (**B**) parietal alpha power (8–13 Hz), and (**C**) frontoparietal alpha synchronization. Note that all instances of the RCS, alpha power, and frontoparietal alpha synchronization are given relative to the Baseline data (i.e., values after subtracting the corresponding Baseline values). Error bars represent the SEM. The underlying data supporting S12 Fig can be found in the Supporting information as S1 Data. RCS, rate correct score; SEM, standard error of the mean; tACS, transcranial alternating current stimulation.
(TIF)

S13 Fig The phase-dependent tACS effects calculated from the combination of Experiment 1 and Experiment 2. When the results from Experiment 1 and the results from the 7-letter trials of Experiment 2 were combined together, anti-phase tACS significantly decreased (**A**) WM performance, (**B**) parietal alpha power, and (**C**) frontoparietal alpha synchronization as compared to in-phase tACS. Note that all instances of the RCS, alpha power, and frontoparietal alpha synchronization are given relative to the Baseline data (i.e., values after subtracting the corresponding Baseline values). Within-group comparisons used two-tailed permuted paired *t*-tests. Error bars represent the SEM; *significant at *p* < 0.05, **significant at *p* < 0.01. The underlying data supporting S13 Fig can be found in the Supporting information as S1 Data. RCS, rate correct score; SEM, standard error of the mean; tACS, transcranial alternating current stimulation.
(TIF)

S1 Data Summary data for all figures. (XLSX)

S1 Table The specifications of the custom designed tACS stimulator. (TIF)

S1 Text Supplementary information. Contains detailed methods for online phase-corrected tACS system, data analysis, and Experiments 2–4.
(DOCX)

## Abbreviations

EEG: electroencephalogram
FFT: fast Fourier transform
IAF: individual alpha frequency
NIBS: noninvasive brain stimulation
PLI: phase lag index
RCS: rate correct score
RT: reaction time
rTMS: repetitive transcranial magnetic stimulation
tACS: transcranial alternating current stimulation
WM: working memory
