# Supplementary figures and images for: Alpha oscillatory activity is causally linked to working memory retention
Source: PLoS Biol. 2023 Feb 13;21(2):e3001999. doi: 10.1371/journal.pbio.3001999 (PMC9983870; doi:10.1371/journal.pbio.3001999)

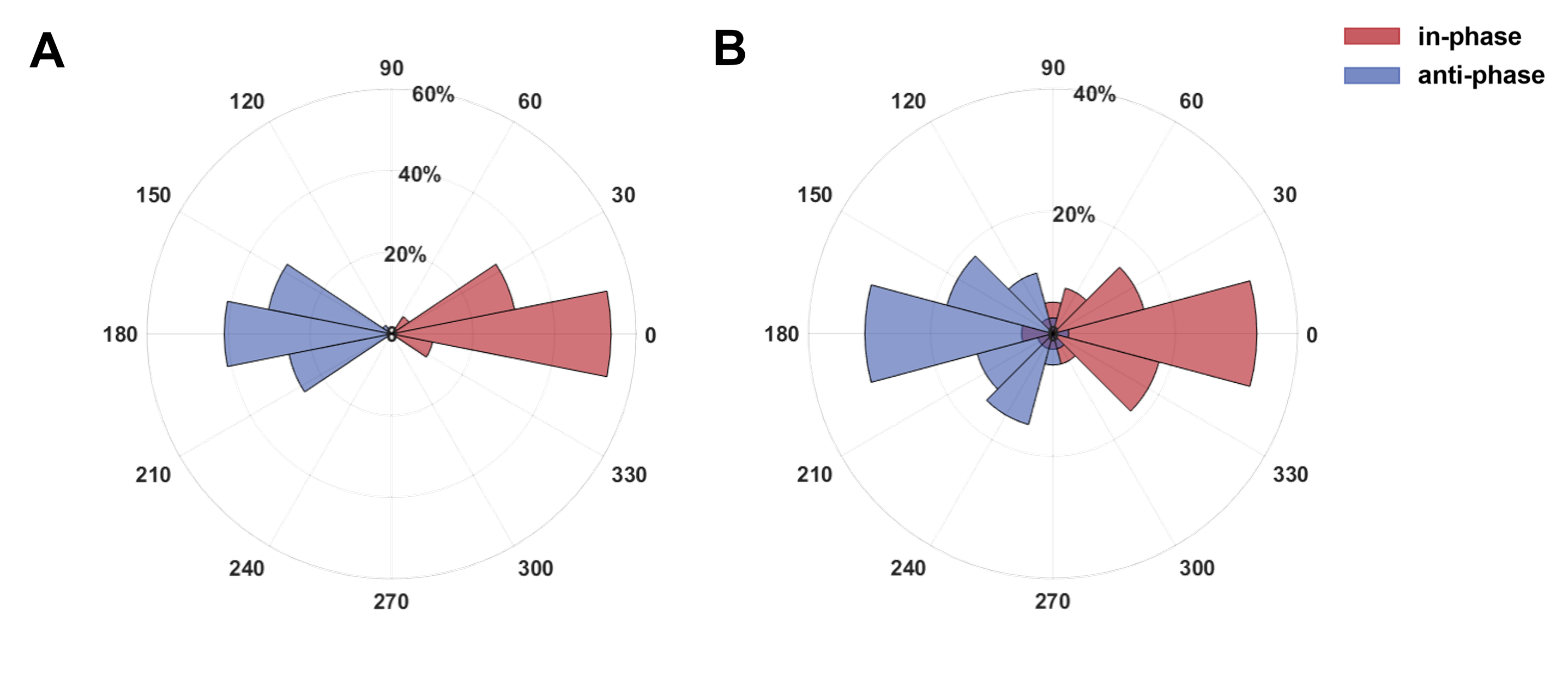

Supplement: S2 Fig — (A) Within the first 0.1 s, the mean and the standard deviation of the phase differences between EEG signals and tACS waveforms was 7.36° ± 15.09° for in-phase tACS and 177.52° ± 15.45° for anti-phase tACS. The phase differences are binned (width, 22.5°) and frequencies are indicated (inner ring = 20%, middle ring = 40%, and outer ring = 60%). (B) During the whole 0.8 s stimulation across all subjects, the mean and the standard deviation of the phase differences between EEG signals and tACS waveforms was 2.42° ± 52.73° for in-phase tACS and 185.65° ± 44.91° for anti-phase tACS. The phase differences are binned (width, 30°) and frequencies are indicated (inner ring = 20%, outer ring = 40%). The underlying data supporting S2 Fig can be found in the Supporting information as S1 Data. EEG, Electroencephalogram; tACS, transcranial alternating current stimulation. (TIF) [file pbio.3001999.s002.tif]

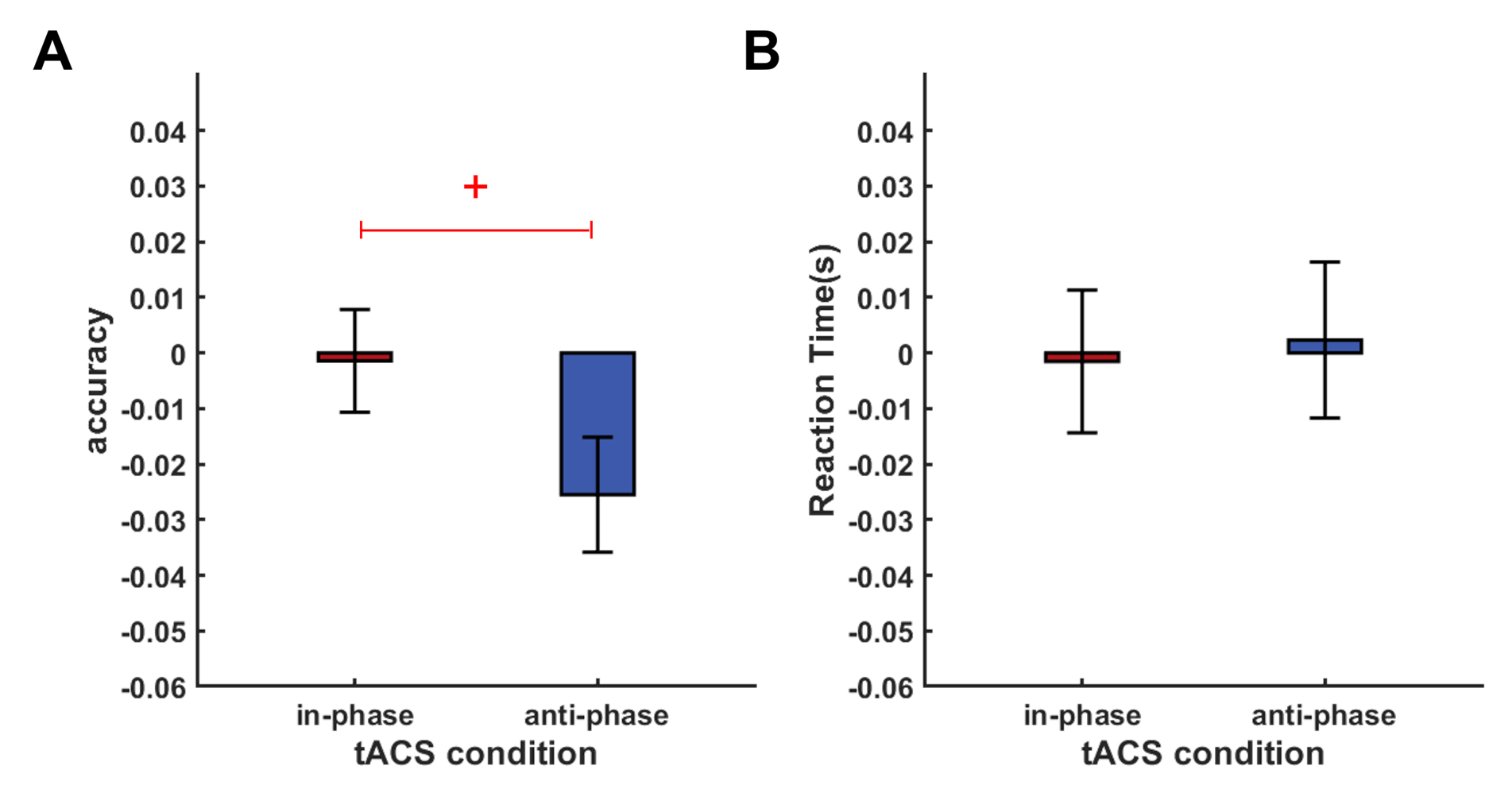

Supplement: S3 Fig — The Online effects of tACS on (A) accuracy and (B) reaction time for the 2 stimulation conditions: in-phase and anti-phase tACS. Accuracy and reaction time are given relative to Baseline. Error bars represent SEM; + marginally significant at 0.05 < p < 0.1. The underlying data supporting S3 Fig can be found in the Supporting information as S1 Data. SEM, standard error of the mean; tACS, transcranial alternating current stimulation. (TIF) [file pbio.3001999.s003.tif]

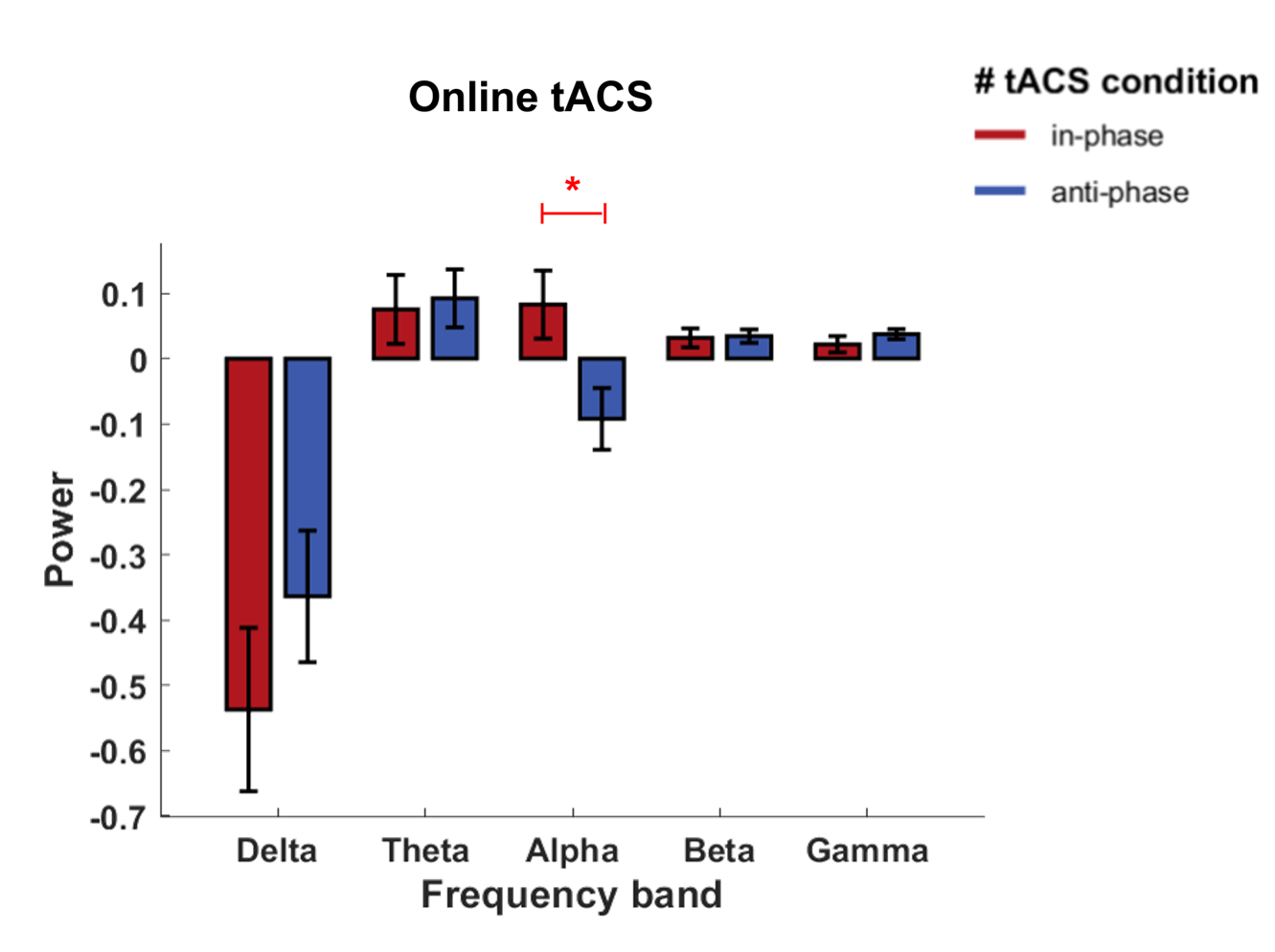

Supplement: S4 Fig — Powers are given relative to the corresponding values at Baseline. Error bars represent the SEM; *significant at p < 0.05, **significant at p < 0.01. The underlying data supporting S4 Fig can be found in the Supporting information as S1 Data. SEM, standard error of the mean; tACS, transcranial alternating current stimulation. (TIF) [file pbio.3001999.s004.tif]

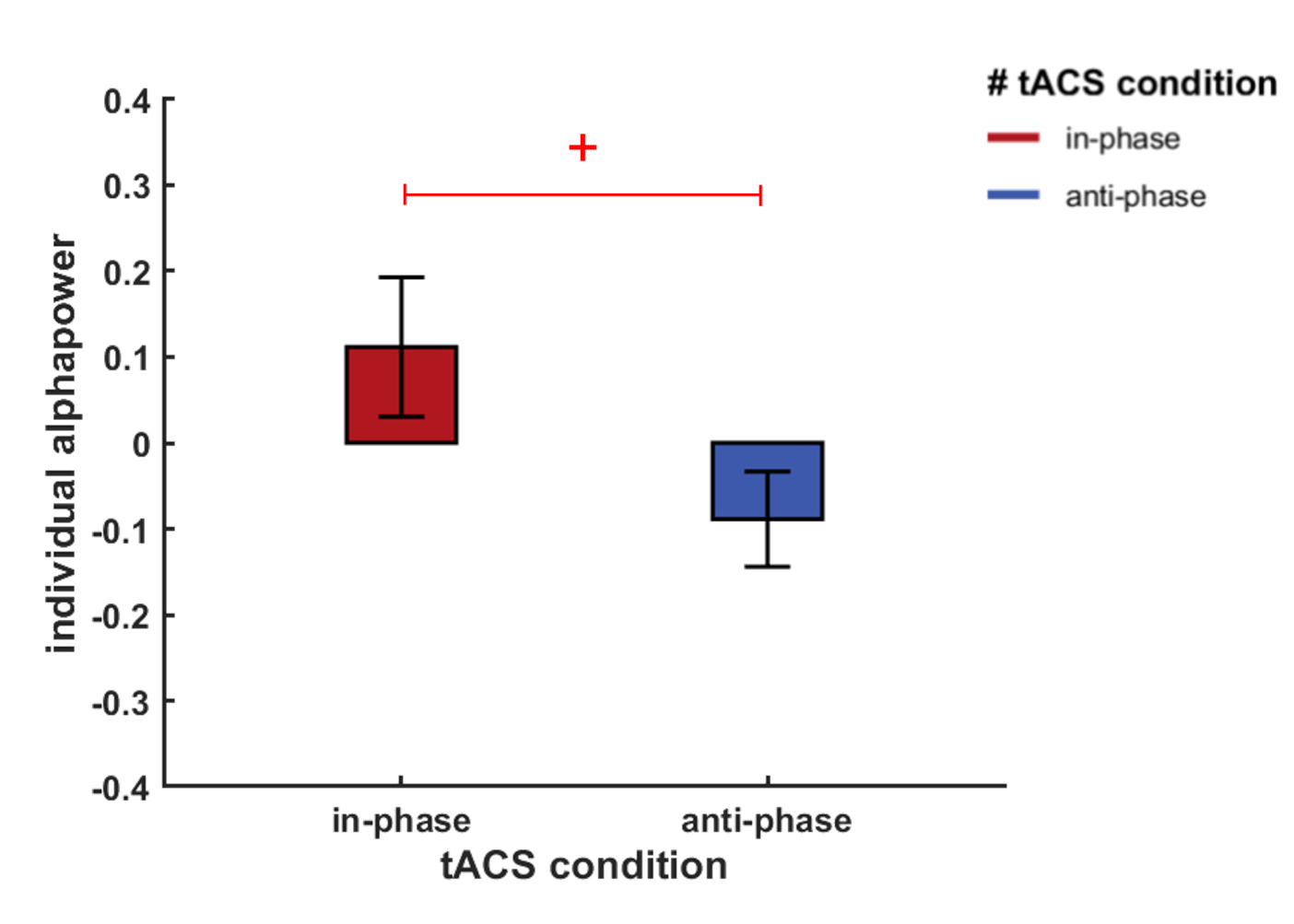

Supplement: S5 Fig — Individual alpha power is given relative to Baseline. Error bars represent SEM; + marginally significant at 0.05 < p < 0.1. The underlying data supporting S5 Fig can be found in the Supporting information as S1 Data. SEM, standard error of the mean; tACS, transcranial alternating current stimulation. (TIF) [file pbio.3001999.s005.tif]

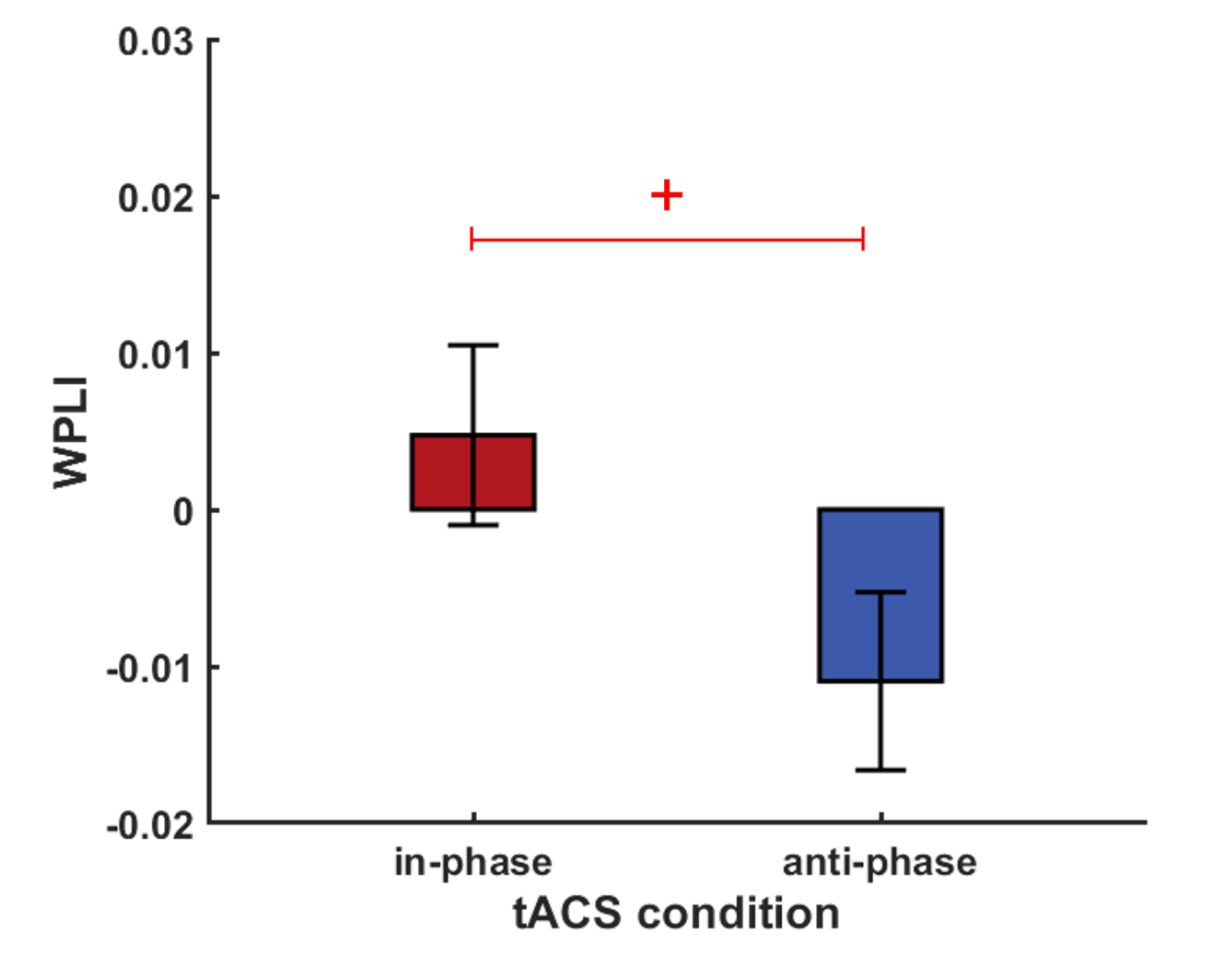

Supplement: S6 Fig — WPLI is given relative to Baseline. Error bars represent SEM; + marginally significant at 0.05 < p < 0.1. The underlying data supporting S6 Fig can be found in the Supporting information as S1 Data. SEM, standard error of the mean; tACS, transcranial alternating current stimulation; WPLI, weighted phase lag index. (TIF) [file pbio.3001999.s006.tif]

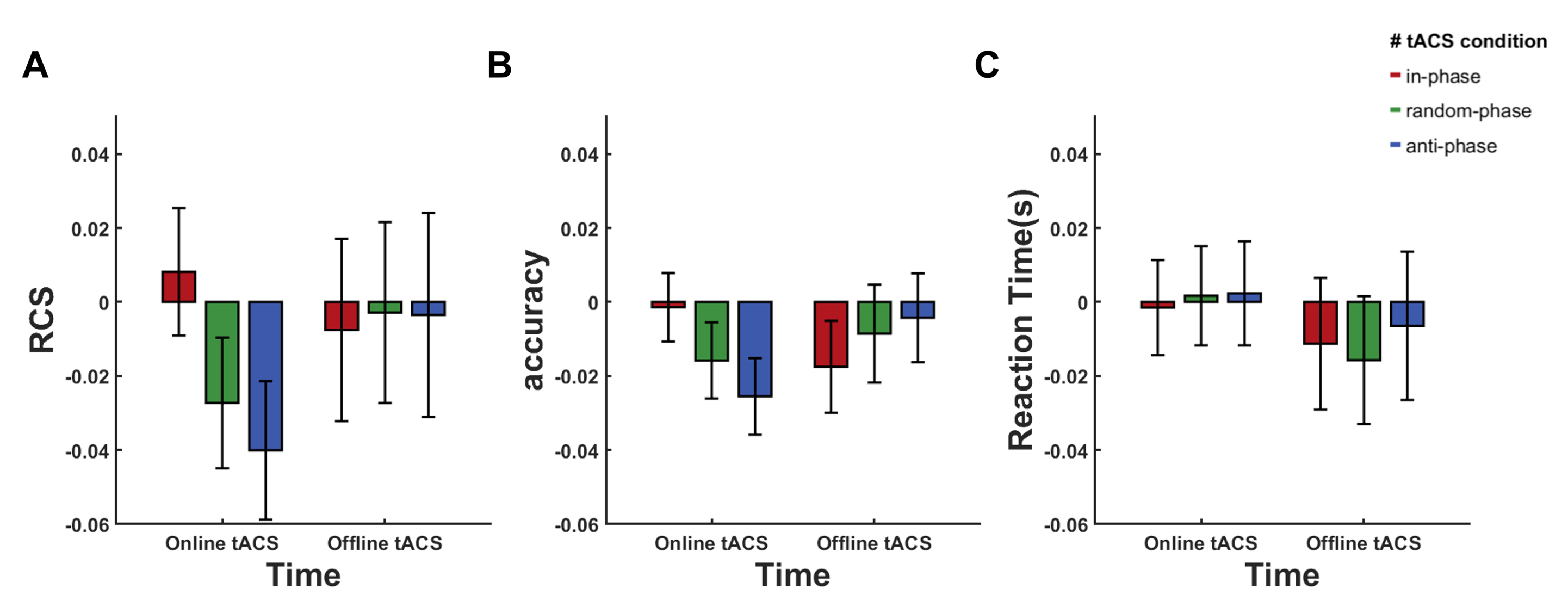

Supplement: S7 Fig — The change for (A) RCS, (B) accuracy, and (C) reaction time during Online tACS and Offline tACS for the 3 stimulation conditions: in-phase, random-phase, and anti-phase tACS. RCS, accuracy, and reaction time are given relative to Baseline (subtract corresponding Baseline values). Error bars represent SEM. The underlying data supporting S7 Fig can be found in the Supporting information as S1 Data. RCS, rate correct score; SEM, standard error of the mean; tACS, transcranial alternating current stimulation. (TIF) [file pbio.3001999.s007.tif]

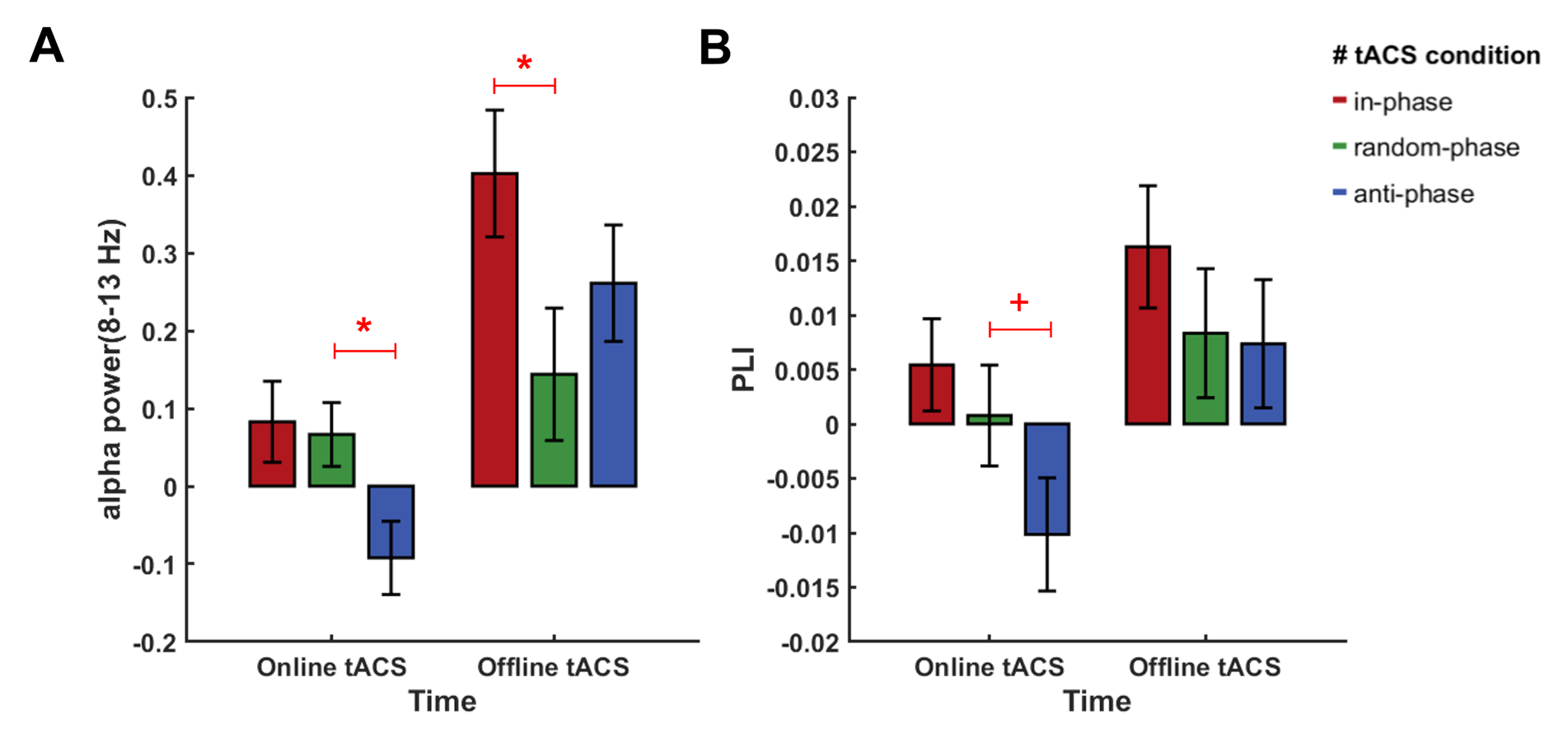

Supplement: S8 Fig — (A) The alpha power of Pz electrode in in-phase and anti-phase tACS compared with random-phase tACS. (B) The frontoparietal alpha synchronization, indexed by PLI in in-phase and anti-phase tACS compared with random-phase tACS. Alpha power and frontoparietal alpha synchronization are given relative to Baseline (subtract corresponding Baseline values). Error bars represent SEM; + marginally significant at 0.05 < p < 0.1, *significant at p < 0.05. The underlying data supporting S8 Fig can be found in the Supporting information as S1 Data. EEG, electroencephalogram; PLI, phase lag index; SEM, standard error of the mean; tACS, transcranial alternating current stimulation. (TIF) [file pbio.3001999.s008.tif]

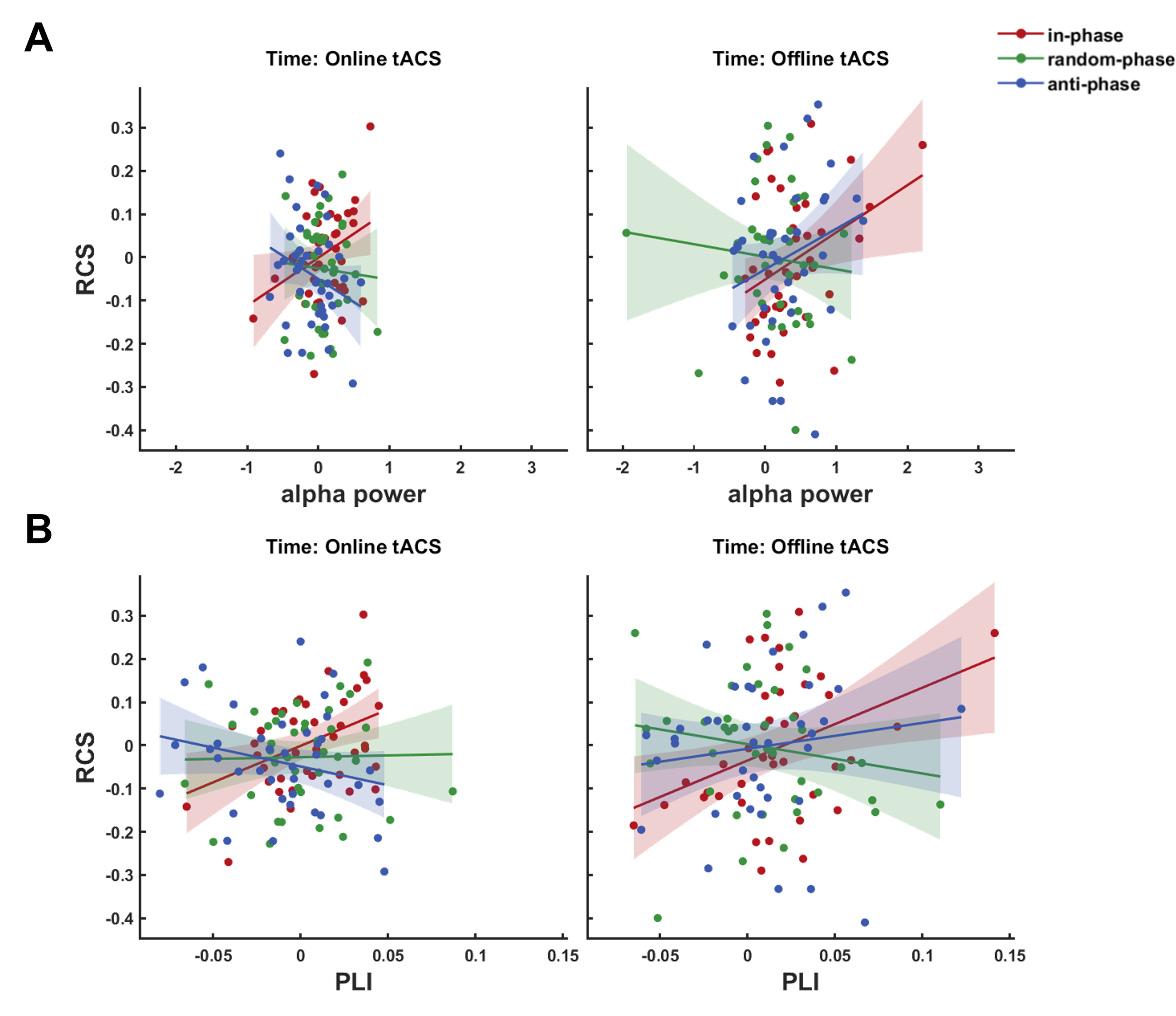

Supplement: S9 Fig — (A) Correlation between the changes in RCS and parietal alpha power relative to Baseline at each time point (Online tACS and Offline tACS). (B) Correlation between the changes in RCS and frontoparietal alpha synchronization at each time point (Online tACS and Offline tACS). RCS, alpha power, and frontoparietal alpha synchronization are given relative to Baseline (subtract corresponding Baseline values). The underlying data supporting S9 Fig can be found in the Supporting information as S1 Data. RCS, rate correct score; SEM, standard error of the mean; tACS, transcranial alternating current stimulation. (TIF) [file pbio.3001999.s009.tif]

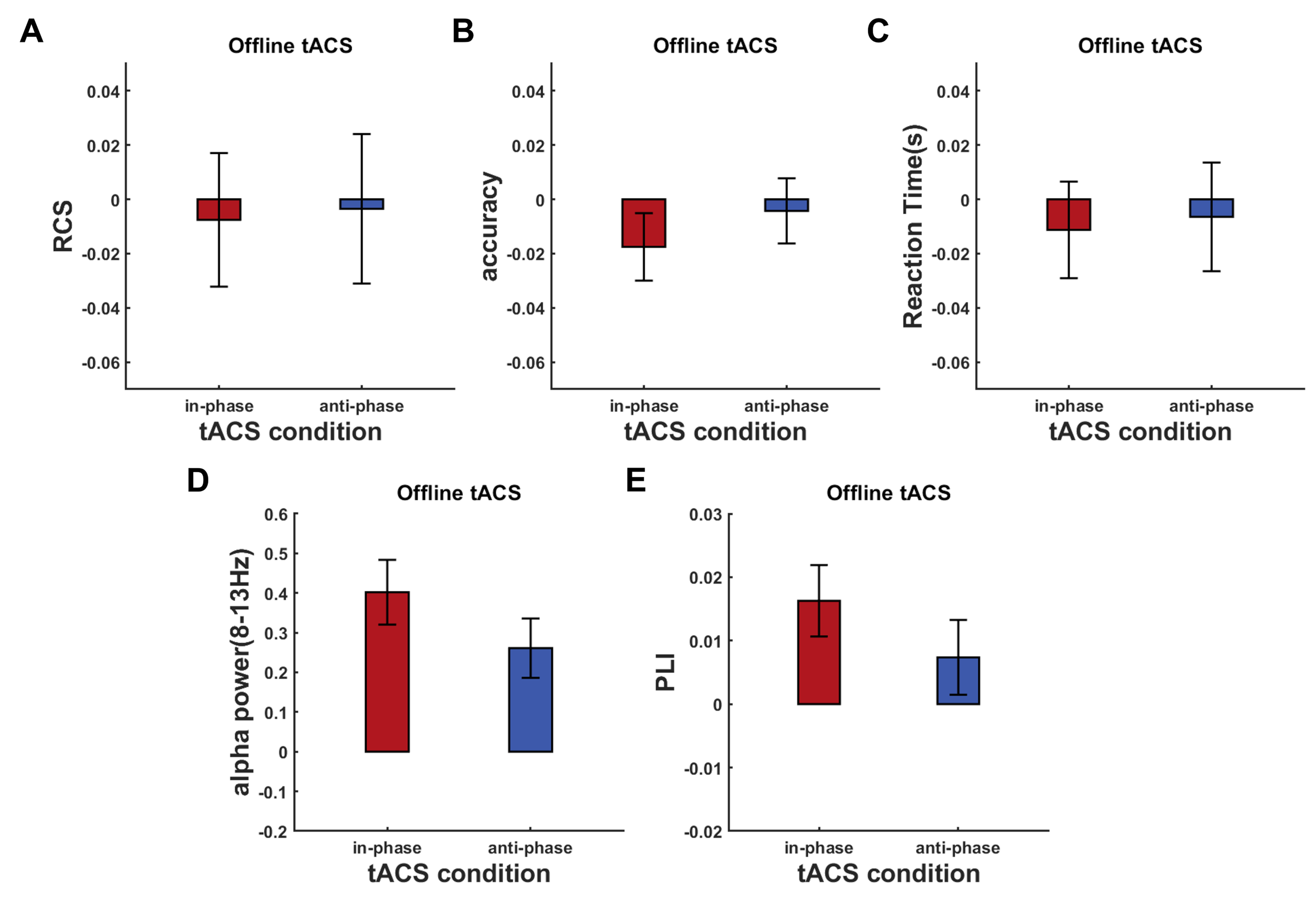

Supplement: S10 Fig — The Offline effects induced by in-phase tACS and anti-phase tACS in (A) RCS, (B) accuracy, (C) RT, (D) alpha power, and (E) frontoparietal alpha synchronization. RCS, accuracy, reaction time, alpha power, and frontoparietal alpha synchronization are given relative to Baseline (subtract corresponding Baseline values). Error bars represent SEM. The underlying data supporting S10 Fig can be found in the Supporting information as S1 Data. RCS, rate correct score; RT, reaction time; SEM, standard error of the mean; transcranial alternating current stimulation. (TIF) [file pbio.3001999.s010.tif]

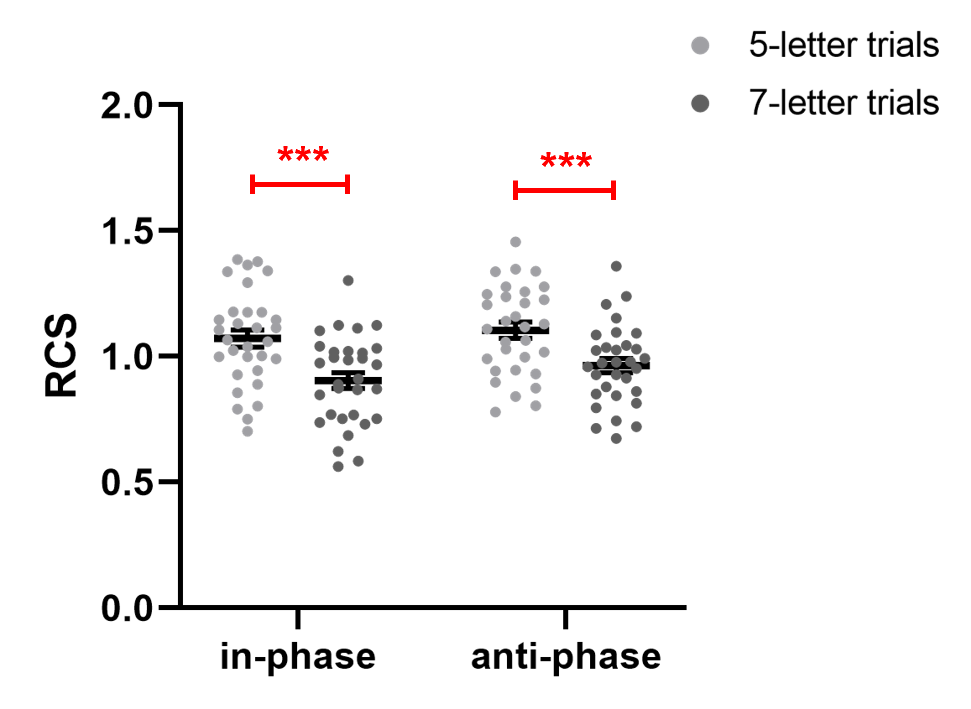

Supplement: S11 Fig — Note that RCS values are original values. Error bars represent the SEM; ***significant at p < 0.001 (two-tailed permuted paired t-tests). The underlying data supporting S11 Fig can be found in the Supporting information as S1 Data. RCS, rate correct score; SEM, standard error of the mean. (TIF) [file pbio.3001999.s011.tif]

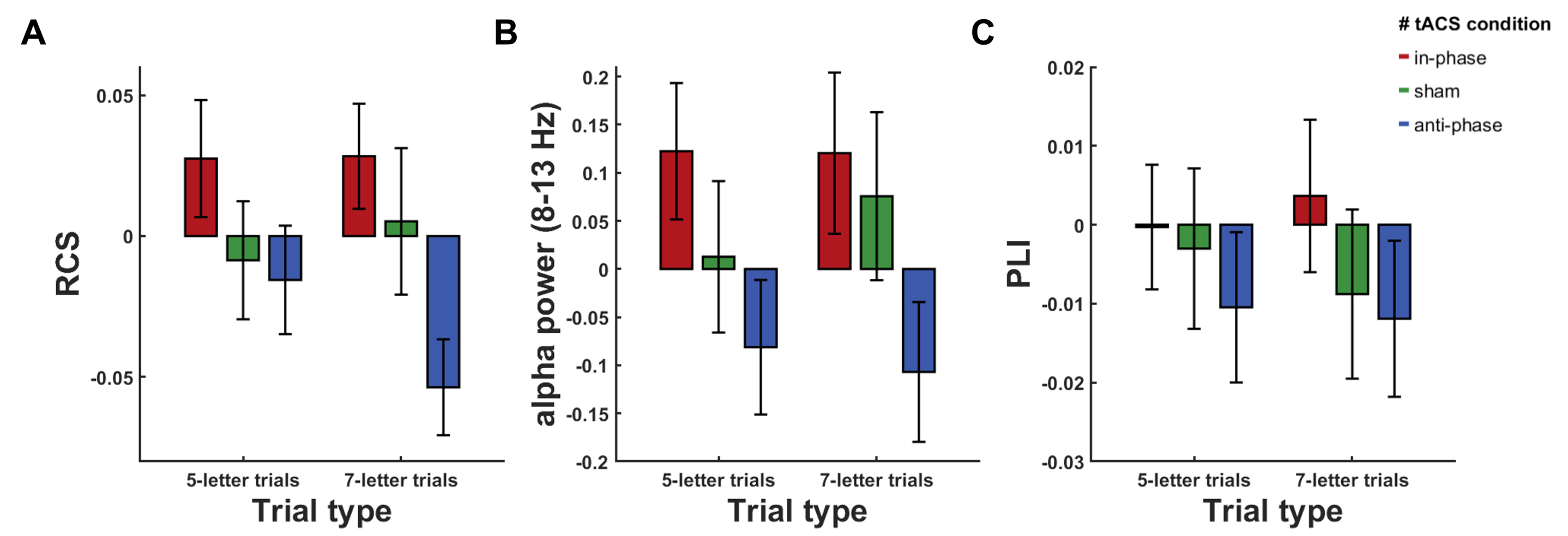

Supplement: S12 Fig — The effects of sham condition were all intermediate between the in-phase tACS and anti-phase tACS for (A) WM performance, (B) parietal alpha power (8–13 Hz), and (C) frontoparietal alpha synchronization. Note that all instances of the RCS, alpha power, and frontoparietal alpha synchronization are given relative to the Baseline data (i.e., values after subtracting the corresponding Baseline values). Error bars represent the SEM. The underlying data supporting S12 Fig can be found in the Supporting information as S1 Data. RCS, rate correct score; SEM, standard error of the mean; tACS, transcranial alternating current stimulation. (TIF) [file pbio.3001999.s012.tif]

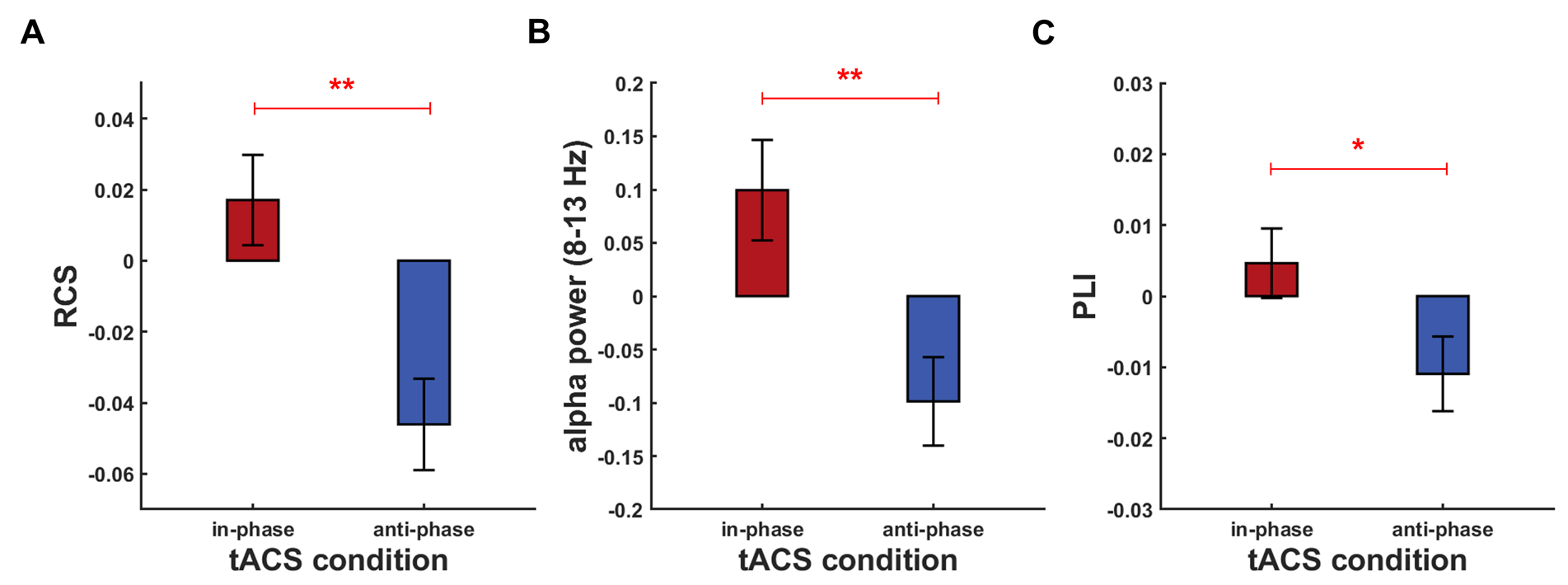

Supplement: S13 Fig — When the results from Experiment 1 and the results from the 7-letter trials of Experiment 2 were combined together, anti-phase tACS significantly decreased (A) WM performance, (B) parietal alpha power, and (C) frontoparietal alpha synchronization as compared to in-phase tACS. Note that all instances of the RCS, alpha power, and frontoparietal alpha synchronization are given relative to the Baseline data (i.e., values after subtracting the corresponding Baseline values). Within-group comparisons used two-tailed permuted paired t-tests. Error bars represent the SEM; *significant at p < 0.05, **significant at p < 0.01. The underlying data supporting S13 Fig can be found in the Supporting information as S1 Data. RCS, rate correct score; SEM, standard error of the mean; tACS, transcranial alternating current stimulation. (TIF) [file pbio.3001999.s013.tif]
