# Supplementary material for: Alpha oscillatory activity is causally linked to working memory retention
Source: PLoS Biol. 2023 Feb 13;21(2):e3001999. doi: 10.1371/journal.pbio.3001999 (PMC9983870; doi:10.1371/journal.pbio.3001999)
Supplement: S1 Text — Contains detailed methods for online phase-corrected tACS system, data analysis, and Experiments 2–4. (DOCX) [file pbio.3001999.s016.docx]

Supplementary Information for

**Alpha oscillatory activity is causally linked to working memory retention**

**Contents**

[**1. More details for Materials and Methods**](#_Toc117804032)

[**1.1. The details of online phase-corrected tACS system**](#_Toc117804033)

[**1.1.1. Signal processing**](#_Toc117804034)

[**1.1.2. Delay correction**](#_Toc117804035)

[**1.1.3. Design of tACS stimulator**](#_Toc117804036)

[**1.2. IAF and threshold determination**](#_Toc117804037)

[**1.3. No systematic fluctuation of alpha frequency with time for the Sternberg WM task**](#_Toc117804038)

[**1.4. Analysis of the phase alignment between EEG signals and tACS waveforms**](#_Toc117804039)

[**2. The comparison of accuracy and RT between in-phase tACS and anti-phase tACS**](#_Toc117804040)

[**3. Supplementary EEG power analysis**](#_Toc117804041)

[**3.1. The comparison of power in full frequency band between in-phase alpha-tACS and anti-phase alpha-tACS**](#_Toc117804042)

[**3.2. The comparison of individual alpha power between in-phase tACS and anti-phase tACS**](#_Toc117804043)

[**4. The comparison of WPLI between in-phase tACS and anti-phase tACS**](#_Toc117804044)

[**5. Compared to Baseline, anti-phase tACS significantly decreased WM performance and alpha power during Online tACS**](#_Toc117804045)

[**6. Complementary analysis of the behavior and EEG data in random-phase tACS**](#_Toc117804046)

[**6.1. The behavioral effects of random-phase tACS compared with in-phase and anti-phase tACS**](#_Toc117804047)

[**6.2. The EEG results of random-phase tACS compared with in-phase and anti-phase tACS.**](#_Toc117804048)

[**6.3. The correlations between random-phase tACS induced changes in behavioral performance and alpha activities**](#_Toc117804049)

[**7. The Offline comparison between in-phase tACS effects and anti-phase tACS effects.**](#_Toc117804050)

[**8. Experiment 2**](#_Toc117804051)

[**8.1. Participants**](#_Toc117804052)

[**8.2. Experimental procedures, stimulation paradigm, and EEG analysis**](#_Toc117804053)

**8.3. The 7-letter trials were difficult than 5-letter trials**

[**8.4. The effects of sham condition were intermediate between the in-phase tACS and anti-phase tACS**](#_Toc117804054)

[**8.5. Sham stimulation caused the problem of blind breaking**](#_Toc117804055)

[**9. The phase-dependent tACS effects were detected from the combined data of Experiment 1 and Experiment 2**](#_Toc117804056)

[**10. Experiment 3**](#_Toc117804057)

[**10.1. Participants**](#_Toc117804058)

[**10.2. Experimental procedures, stimulation paradigm, and EEG analysis**](#_Toc117804059)

[**10.3. The effects of theta tACS differed from alpha tACS**](#_Toc117804060)

[**10.4. Blinding**](#_Toc117804061)

[**11. Experiment 4**](#_Toc117804062)

[**11.1. Participants**](#_Toc117804063)

[**11.2. Experimental procedures, stimulation paradigm, and EEG analysis**](#_Toc117804064)

[**11.3. Blinding**](#_Toc117804065)

[**12. Supplementary discussion**](#_Toc117804066)

[**12.1. A discussion regarding positioning of the stimulation electrode**](#_Toc117804067)

[**12.2. A discussion on the non-significance of in-phase tACS-induced enhancement effects**](#_Toc117804068)

# 1. More details for Materials and Methods

## 1.1. The details of online phase-corrected tACS system

Illustrated in **Fig 1**, the online phase-corrected tACS system included an EEG instrument, computation module, and a custom designed tACS stimulator which could receive the instructions from the computation module. The phase-corrected brain stimulation algorithm for online phase-corrected tACS was introduced that could record brain oscillations by an EEG instrument, analyze the raw data online by computational algorithms to extract the phase and amplitude of underlying brain rhythms, deliver tACS with a desired phase difference relative to the endogenous targeted brain oscillations based on the computational outcome, and closing the loop, affect the neuronal activity.

### 1.1.1. Signal processing

The EEG signal of interested electrode (Pz electrode for our study) was stored in a moving 500 ms time window. Because of the performance setting of EEG amplifier, the EEG data was updated every 10ms, and the analysis should be finished within this time range. First, the recorded EEG signal over the time window was bandpass filtered to extract the activity within the frequency range of interest (IAF ± 2Hz for alpha-tACS). A 200th order finite impulse response (FIR) filter was used because of its linear-phase property, and the raw data was reversed before filter to maintain the latest data after phase correction. Then, whether there were peaks or troughs in the last 10ms of the filtered signal were estimated based on the fact that the peaks and troughs are local maximums or minimums.

In in-phase tACS, stimulation was triggered when two consecutive peaks exceeded the threshold (in our study, threshold was calculated previously using the EEG signal at Baseline, see more details in “1.2 IAF and threshold determination”); while in the condition of anti-phase tACS, the stimulation was triggered when the absolute value of two consecutive troughs exceeded the threshold. In theory, in both in-phase and anti-phase tACS, the algorithm waited for a time interval of 3/4 cycles at the frequency of interest (IAF for our study) when the pre-determined requirements to trigger tACS were satisfied, and then the stimulation was applied to the participants by the tACS stimulator. To correct for the time delays in the system introduced by the signal processing or hardware delays, the delay time was experimentally measured and corrected (see more details in “1.1.2. Delay correction” section). The onset phase of the sine-wave stimulation was invariably 0 in all conditions. As a result, the phase difference between in-phase tACS and the endogenous alpha activity was 0°, while the phase difference between anti-phase tACS and the endogenous alpha activity was 180°.

In random-phase tACS, to make the phase difference not fixed across trials, we artificially set whether to apply tACS during the retention sub-process of a certain trial and the start time of tACS within each trial. 5 participants were recruited to complete a pretest in which every participant performed two stimulation sessions: in-phase tACS and anti-phase tACS. Their stimulation sequences (whether to apply tACS in each trial) and the start times of tACS within each trial (the start time of tACS relative to the onset of the retention sub-process if stimulation was triggered in a trial) were collected. When a participant performed random-phase session in the formal experiment, we randomly assigned one of the 10 stimulation sequences to the participant. Then if tACS should be applied in a trial, we specified the start time of tACS within the WM retention sub-process of the trial by randomly selecting one of the start times of tACS collected in the pretest.

### 1.1.2. Delay correction

In in-phase and anti-phase tACS, when the alpha activity reached the threshold for triggering tACS, the application time of tACS stimulation should be precisely controlled so that the phase difference between the tACS waveform and the endogenous alpha oscillations was 0° or 180°. However, it should be noted that signal processing components, such as the hardware filters used in EEG recording instruments, and the software filters applied to the signal during processing, could introduce frequency or cycle time dependent delays to the recordings. Additionally, hardware delays in communicating the stimulation instructions to the stimulator were also inevitable.

We addressed this issue by experimentally measuring the time delays introduced by all the components of the system, and then applying a time correction to achieve the desired phase difference between tACS waveform and the endogenous brain oscillations. To obtain and calibrate the delays, we used a 50Ω resistor to simulate the resistance of human brain and a signal generator (XD2, Zhonghuan Keyi Electronic Instrument Co., Ltd, Tianjin, China) to simulate the EEG signal generated by the brain. We then started the online phase-corrected tACS system, connected the output of the signal generator and the output of the tACS stimulator to the EEG recording instrument, and then analyzed the collected data to obtain the delays of the tACS waveform relative to the simulated EEG signal.

We randomly selected 10 frequencies that may be used in the formal experiments within the frequency range of 8-13 Hz and tested the time delays in both in-phase and anti-phase tACS conditions. For each frequency, we applied online phase-corrected tACS stimulation at that frequency with a duration of 0.5s for 100 times, and recorded the tACS waveform and the sinusoidal waveform at the same frequency generated by the signal generator continuously. The voltage of tACS was about 8.75mV, lower than the saturation voltage of the EEG amplifier. We then computed the delays between the first peak of each tACS waveform segment and the nearest peak of signals generated by the signal generator in in-phase tACS, or computed the delays between the first peak of each tACS waveform segment and the nearest through of the simulated EEG signals in anti-phase tACS, which was actually the system delays.

At the same frequency, there was no difference between the system delays measured in in-phase tACS and anti-phase tACS. Furthermore, we found that the delays were positively correlated with the cycle time of the tACS waveform (r = 0.999, p <0.001) (**S1 Fig**). The delays could be estimated from the formula: L = 1.2013*(1000/f)-34.8, where L was the delays and f was the tACS frequency. The unit of L was milliseconds, and 1000/f represented the cycle time of the tACS waveform at the frequency of f Hz. In the formal experiment, we used the formula above to estimate the system delays for a given tACS frequency and subtracted the delays from the waiting time when tACS was triggered. After delay correction, the system delays measured in in-phase tACS and anti-phase tACS were 0 ms.

### 1.1.3. Design of tACS stimulator

To apply tACS dependent on the brain states, we designed a tACS stimulator which could communicate with the computer through serial USB. To protect participants from any potential harm, voltages of the output port were monitored by protect circuits to ensure security. Once the voltage exceeded the safety threshold (36 V, peak to peak), the output port would be cut down by the relay. The tACS stimulator is powered by four rechargeable batteries and isolated from mains electricity. The computer communicating with the tACS stimulator also stopped charging once the phase-corrected tACS was connected with participants.

Illustrated in **Fig 1B**, the main part of the stimulator consisted of an Arduino Uno microcontroller board, a digital-to-analog converter and two operational amplifiers. The Arduino Uno microcontroller board was the core control part of our designed stimulator. The timing of the next tACS calculated by the computer was communicated to an Arduino Uno Microcontroller board (Arduino) through serial USB communication. Then, the stimulator follows the Arduino-generated voltage waveforms and produces a current controlled output proportional to that voltage.

The specifications of the custom designed tACS stimulator were illustrated in **S1 Table**.

## 1.2. IAF and threshold determination

To determine the IAF and the threshold for triggering tACS, the EEG data at Baseline was analyzed. The first 0.5s EEG data in each trial at Baseline was discarded due to the weak alpha oscillations, and the remaining EEG data at Pz electrode was analyzed. To this end, there were sixty 2s segments. Subsequently, the segments corresponding to the error trials were deleted. Preprocessing included the following steps: detrending, blink artifacts correction (using independent component analysis approach), rejecting all segments containing activity exceeding a threshold of 100 uV, and the removal of segments with residual eye blinks or other artifacts. After preprocessing, a fast Fourier transformation (FFT, frequency resolution 0.1953 Hz) was performed for each remaining segment, and the resulting spectra were averaged. The prominent alpha peak (8-13 Hz) was detected and its frequency was defined as IAF.

To really target the interested endogenous oscillations (i.e., alpha oscillations), we aimed to deliver tACS only when strong endogenous interested oscillations exist as previous brain state-dependent tACS studies did [1-3]. Therefore, we calculated a threshold for triggering tACS. We performed an offline analysis of the Baseline EEG data recorded in the retention sub-process of each trial the same as the online EEG monitoring (see more details in Supplementary material: 1.1.1. Signal processing). The window length was 500ms and the sliding step was 10ms. Bandpass filtering was performed on the EEG signals in each time window to extract the activity within the frequency range of IAF ± 2Hz. Then we decided whether a peak or trough existed in the last 10 ms of the alpha oscillations extracted from each time window, and recorded the detected peak or trough values. The lower quartile of all peak values and the lower quartile of all absolute values of troughs were used as the threshold for in-phase tACS and anti-phase tACS, respectively.

## 1.3. No systematic fluctuation of alpha frequency with time for the Sternberg WM task

To examine whether there was a mismatch between tACS frequency and endogenous alpha frequency that results from the systematic change in alpha frequency over the course of a task [4], we compared participants’ IAF at Baseline (i.e., tACS frequency) with their IAF during Online tACS.

We calculated participants’ IAF during Online tACS using the same method as the Baseline. Permuted paired *t*-tests revealed that there were no differences between IAF at Baseline and IAF during Online tACS for the in-phase, random-phase, or anti-phase tACS (in-phase: t = 1.70, p = 0.10; random-phase: t = 1.34, p = 0.19; anti-phase: t = 0.69, p = 0.49). These results demonstrated that there were no systematic fluctuations in IAF with the time for the Sternberg WM task. Thus, the use of Baseline IAF as the tACS frequency does not result in a systematic bias between the tACS frequency and the endogenous alpha frequency.

## 1.4. Analysis of the phase alignment between EEG signals and tACS waveforms

As the EEG signals were not recorded during the simultaneous applications of 0.8 s tACS, the accuracy of the phase alignment between tACS waveforms and EEG signals cannot be assessed during Online tACS. To assess the accuracy of the phase-corrected tACS system, we exploited the Baseline EEG data to simulate the artificial 0.8 s tACS waveforms offline using the tACS trigger algorithm same as Online tACS, and then calculated the phase alignment between EEG signals and tACS waveforms in the first 0.1 s and throughout the 0.8 s. The EEG signals recorded at Baseline were bandpass filtered to extract alpha oscillations. The phases of filtered EEG (φEEG) and tACS waveforms (φtACS) were extracted with Hilbert transformation. Then the phase differences between EEG signals and tACS waveforms were calculated using the following formula: phase differences = angle(mean(exp(i*(φEEG-φtACS)))). The item exp(i*(φEEG-φtACS)) was averaged across time points within a single trial and then averaged across trials within a single experiment session.

To examine whether the phase differences between EEG signals and tACS waveforms were 0° for the in-phase tACS and 180° for the anti-phase tACS, analysis of the phase differences was performed using the Circular Statistics Toolbox [5]. Within the first 0.1 s, the mean and the standard deviation of the phase differences between EEG signals and tACS waveforms was 7.36° ± 15.09° for the in-phase tACS and was 177.52° ± 15.45° for the anti-phase tACS (**S2A Fig**). Within the 0.8 s, the mean and the standard deviation of the phase differences between EEG signals and tACS waveforms was 2.42° ± 52.73° for the in-phase tACS and was 185.65° ± 44.91° for the anti-phase tACS (**S2B Fig**). The Rayleigh test for circular uniformity indicated that the phase differences between EEG signals and tACS waveforms were not uniformly distributed for either in-phase tACS (the first 0.1 s: p < 0.001; 0.8 s: p < 0.001) or anti-phase tACS (the first 0.1 s: p < 0.001; 0.8 s: p < 0.001). The Watson-Williams test revealed that the phase differences between EEG signals and tACS waveforms in the in-phase tACS were significantly different from that in the anti-phase tACS (the first 0.1 s: p < 0.001; 0.8 s: p < 0.001). These findings support the accuracy of the phase-corrected tACS system correcting for the phase differences between EEG signals and tACS waveforms.

# 2. The comparison of accuracy and RT between in-phase tACS and anti-phase tACS

In Experiment 1, we used paired *t*-tests to explore whether anti-phase tACS induced lower accuracy and slower response time compared to in-phase tACS during stimulation, as we hypothesized that anti-phase tACS impaired WM performance compared to in-phase tACS. Compared to in-phase tACS, anti-phase tACS induced a marginally significant reduction in accuracy (t(38) = -1.958, p = 0.057, Cohen’s d = 0.314; permuted paired *t*-test) (**S3A Fig**), which further supports the different modulation effects of in-phase tACS and anti-phase tACS on WM performance. No significant difference in RT (t(38) = -0.21, p = 0.840; permuted paired *t*-test) was found between in-phase tACS and anti-phase tACS (**S3B Fig**).

# 3. Supplementary EEG power analysis

## 3.1. The comparison of power in full frequency band between in-phase alpha-tACS and anti-phase alpha-tACS

Many previous studies have shown the frequency-specific modulation of tACS in both animals [6] and humans [7]. To further support the modulation effects of our online phase-corrected tACS on brain activity, we tested the influences of this innovative tACS system on the full physiological frequency band of the EEG (from 1 Hz to 45 Hz). By convention, the waking EEG was divided into five distinct frequency bands: delta (1-4Hz), theta (4-8Hz), alpha (8-13Hz), beta (13-30Hz) and gamma (30-45Hz). We computed the relative power of each frequency band, with the analysis method same as alpha power (See detailed information in Methods). Permuted paired *t*-tests were performed to compare the differences between in-phase and anti-phase tACS within each frequency band. No significant differences were found in any of the frequency bands except for the alpha band during Online tACS (delta: t(38) = -1.127, p = 0.271; theta: t(38) = -0.246, p = 0.816; alpha: t(38) = 2.329, p = 0.023; beta: t(38) = -0.141, p = 0.890; gamma: t(38) = -1.073, p = 0.303; permuted paired *t*-tests) (**S4 Fig**). As hypothesized, these results demonstrated that the modulation of the endogenous brain oscillations was restricted to the tACS frequency.

## 3.2. The comparison of individual alpha power between in-phase tACS and anti-phase tACS

Considering that the frequency of our alpha-tACS was calculated based on individual traits (IAF), we also analyzed the impact of alpha tACS on the individual alpha power within the frequency range of IAF ± 2Hz at Pz electrode given that alpha oscillations within this frequency band were monitored during phase-corrected tACS, with the analysis method same as alpha power (8-13 Hz) (See detailed information in Methods). Permuted paired *t*-tests were performed to compare the differences in individual alpha power between in-phase and anti-phase tACS. Anti-phase tACS marginally significantly decreased the alpha power at Pz electrode compared to in-phase tACS during Online tACS (t(38) = -1.976, p = 0.053, Cohen’s d = 0.316; permuted paired *t*-test) (**S5 Fig**). This result is consistent with the result about alpha power (8-13Hz), further supporting the modulation effects of alpha-tACS on alpha oscillations.

# 4. The comparison of WPLI between in-phase tACS and anti-phase tACS

To further illustrate the reliability of our results, we chose another index from the family of phase-synchronization indices, weighted phase lag index (WPLI) [8], to measure frontoparietal alpha synchronization. WPLI may be more sensitive to additional, unrelated noise resources. We found that compared to in-phase tACS, anti-phase tACS marginally significantly disturbed frontoparietal alpha synchronization during Online tACS (t(38) = -1.803, p = 0.075, Cohen’s d = 0.289; permuted paired *t*-test) (**S6 Fig**). This result is consistent with the result calculated by PLI, suggesting that our online phase-corrected tACS modulate the connectivity of distributed brain regions.

# 5. Compared to Baseline, anti-phase tACS significantly decreased WM performance and alpha power during Online tACS

Compared to in-phase tACS, anti-phase tACS induced a significant online down-regulation in WM performance, alpha power, as well as frontoparietal alpha synchronization during Online tACS. To further investigate whether the down-regulation was due to in-phase tACS-induced improvement or anti-phase tACS-induced suppression, we directly compared the original values measured at Baseline with the original values during Online tACS (without subtracting the corresponding Baseline values). RCS and accuracy in anti-phase tACS reduced significantly during Online tACS (RCS: t(38) = -2.117, p = 0.047, Cohen’s d = 0.339; accuracy: t(38) = -2.434, p = 0.019, Cohen’s d = 0.390; permuted paired *t*-tests). Anti-phase tACS also induced marginally significant disturbance in alpha power at the Pz electrode and frontoparietal alpha synchronization during Online tACS (alpha power: t(38) = -1.93, p = 0.058, Cohen’s d = 0.309; PLI: t(38) = -1.926, p = 0.061, Cohen’s d = 0.308; permuted paired *t*-tests). For in-phase tACS, no significant improvement was observed from Baseline to Online tACS for all of the metrics (RCS: t(38) = 0.467, p = 0.642; accuracy: t(38) = -0.152, p = 0.887; RT: t(38) = -0.118, p = 0.909; alpha power: t(38) = 1.568, p = 0.129; PLI: t(38) = 1.266, p = 0.207; permuted paired *t*-tests); the reasons for the non-significant enhancement effects of in-phase tACS may be due to the difficulty to further enhance endogenous strong oscillations by intermittent tACS (See more details in “12.2. A discussion on the non-significance of in-phase tACS-induced enhancement effects”). These results indicated that the different effects between in-phase tACS and anti-phase tACS during Online tACS were mainly due to the suppression effects of anti-phase tACS.

# 6. Complementary analysis of the behavior and EEG data in random-phase tACS

To explore the effects of the control condition, here we compared the effects of random-phase tACS with both in-phase and anti-phase tACS. Permutation tests for one-way repeated-measures analysis of variance (ANOVA) were performed for behavioral performance and electrophysiological metrics, with tACS condition (in-phase tACS, random-phase tACS, and anti-phase tACS) as the within-subject factor. We reshuffled the three values measured at the three tACS conditions randomly within each participant 5000 times to create a distribution of F values, and computed an empirical p value from this distribution.

## 6.1. The behavioral effects of random-phase tACS compared with in-phase and anti-phase tACS

During Online tACS, the main effect of tACS condition was not significant for RCS (F(2,76) = 1.976, p = 0.127), accuracy (F(2,76) = 1.578, p = 0.182) and RT (F(2,76) = 0.027, p = 0.971). Compared with random-phase tACS, either in-phase or anti-phase tACS didn’t show significant differences in RCS (in-phase vs random-phase: t(38) = 1.464, p = 0.146; anti-phase vs random-phase: t(38) = -0.444, p = 0.661; permuted paired *t*-tests), accuracy (in-phase vs random-phase: t(38) = 1.043, p = 0.302; anti-phase vs random-phase: t(38) = -0.659, p = 0.521; permuted paired *t*-tests) and RT (in-phase vs random-phase: t(38) = -0.186, p = 0.853; anti-phase vs random-phase: t(38) = 0.036, p = 0.975; permuted paired *t*-tests) (**S7 Fig**). Despite the lack of significant differences, the three behavioral metrics of random-phase tACS were all between that of in-phase tACS and anti-phase tACS at the stimulation period.

During Offline tACS, the main effect of tACS condition was not significant for RCS (F(2,76) = 0.011, p = 0.986), accuracy (F(2,76) = 0.420, p = 0.551), and RT (F(2,76) = 0.069, p = 0.927). Compared to random-phase tACS, the behavioral effects of in-phase tACS and anti-phase tACS also didn’t reach significance (RCS: in-phase vs random-phase: t(38) = -0.153, p = 0.877; anti-phase vs random-phase: t(38) = -0.017, p = 0.986. accuracy: in-phase vs random-phase: t(38) = -0.608, p = 0.540; anti-phase vs random-phase: t(38) = 0.278, p = 0.782. RT: in-phase vs random-phase: t(38) = 0.215, p = 0.838; anti-phase vs random-phase: t(38) = 0.348, p = 0.722; permuted paired *t*-tests) (**S7 Fig**).

## 6.2. The EEG results of random-phase tACS compared with in-phase and anti-phase tACS.

During Online tACS, permuted one-way repeated-measures ANOVA revealed a significant main effect of tACS condition for both parietal alpha oscillations (F(2,76) = 4.596, p =0.006, *ɳ*_p_^2^ = 0.108) and frontoparietal alpha synchronization (F(2,76) = 2.919, p = 0.049, *ɳ*_p_^2^ = 0.071), supporting the specificity of the phase effects. The alpha power at Pz electrode in random-phase tACS was between in-phase tACS and anti-phase tACS (in-phase vs random-phase: t(38) = 0.291, p = 0.767; anti-phase vs random-phase: t(38) = -2.703, p = 0.012; permuted paired *t*-tests) (**S8A Fig**). The change of frontoparietal alpha synchronization induced by random-phase tACS was also between in-phase tACS and anti-phase tACS during tACS (in-phase vs random-phase: t(38) = 0.747, p = 0.456; anti-phase vs random-phase: t(38) = -1.824, p = 0.079; permuted paired *t*-tests) (**S8B Fig**). These results indicated that the physiological effects of random-phase tACS are also between in-phase and anti-phase tACS as hypothesized despite of the small effect size.

During Offline tACS, permuted one-way repeated-measures ANOVA revealed a significant main effect of stimulation condition for alpha power (F(2,76) = 3.877, p = 0.002, *ɳ*_p_^2^ = 0.093). Permuted paired *t*-tests demonstrated a significant increase of alpha power in in-phase tACS compared with random-phase tACS (in-phase vs random-phase: t(38) = 2.484, p = 0.013), but no significant difference was observed in anti-phase tACS (anti-phase vs random-phase: t(38) = 1.406, p = 0.175) (**S8A Fig**). Results for frontoparietal alpha synchronization showed nonsignificant effects for the main effect of stimulation condition (F(2,76) = 0.736, p = 0.463). Compared to random-phase tACS, the changes of frontoparietal alpha synchronization induced by in-phase tACS and anti-phase tACS were not significant (in-phase vs random-phase: t(38) = 0.961, p = 0.341; anti-phase vs random-phase: t(38) = -0.124, p = 0.906; permuted paired *t*-tests) (**S8B Fig**).

## 6.3. The correlations between random-phase tACS induced changes in behavioral performance and alpha activities

For random-phase tACS, changes in alpha power and RCS were not related during and after tACS (Online tACS: r = -0.06, p = 0.695; Offline tACS: r = -0.101, p = 0.532; permuted Pearson’s correlation). What’s more, the correlation coefficients of random-phase tACS were between that of in-phase tACS and anti-phase tACS, which supports that the positive correlation between alpha power and WM performance was not merely due to the general effects of tACS (**S9A Fig**).

No correlation between changes in frontoparietal alpha synchronization and changes in RCS was observed for random-phase tACS during and after tACS (Online tACS: r = 0.021, p = 0.935; Offline tACS: r = -0.165, p = 0.309; permuted Pearson’s correlation). In consistent with the correlation between changes in alpha power and changes in RCS, the correlation coefficient of random-phase tACS was also between that of in-phase tACS and anti-phase tACS, which supports that not tACS but in-phase tACS induced the increase of frontoparietal alpha synchronization and further helped to improve WM performance (**S9B Fig**).

# 7. The Offline comparison between in-phase tACS effects and anti-phase tACS effects.

Finally, we explored whether anti-phase tACS induced offline suppression in WM performance and alpha activity compared to in-phase tACS using permuted paired *t*-tests. Compared to in-phase tACS, anti-phase tACS didn’t suppress WM performance (RCS: t(38) = 0.123, p = 0.900; accuracy: t(38) = 0.941, p = 0.325; RT: t(38) = 0.178, p = 0.860; permuted paired *t*-tests), parietal alpha power (t(38) = -1.566, p = 0.126; permuted paired *t*-test), or frontoparietal alpha synchronization (t(38) = -1.105, p = 0.284; permuted paired *t*-test) (**S10 Fig**).

Then we further explored the changes from Baseline to Offline tACS within in-phase and anti-phase tACS. Compared to Baseline, no difference in anti-phase tACS was observed during Offline tACS, except for the significant enhancement of parietal alpha power (RCS: t(38) = -0.125, p = 0.898; accuracy: t(38) = -0.351, p = 0.747; RT: t(38) = -0.319, p = 0.754; alpha power: t(38) = 3.444, p = 0.002, Cohen’s d = 0.551; PLI: t(38) = 1.235, p = 0.242; permuted paired *t*-tests). For in-phase tACS, there were also no significant increase in WM performance, but significant increase in alpha power and frontoparietal alpha synchronization (RCS: t(38) = -0.303, p = 0.756; accuracy: t(38) = -1.392, p = 0.154; RT: t(38) = -0.626, p = 0.535; alpha power: t(38) = 4.864, p < 0.001, Cohen’s d = 0.779; PLI: t(38) = 2.86, p= 0.008, Cohen’s d = 0.458; permuted paired *t*-tests). Considering that the enhancement of alpha activities were observed in both in-phase and anti-phase tACS, the significant increase in alpha activities during Offline tACS may be due to the increased efforts needed to suppress irrelevant information after a long time of task performance [9], thereby not contributing to the improvement of WM performance.

# 8. Experiment 2

## 8.1. Participants

35 volunteers participated in Experiment 2. 4 participants were excluded from the analyses due to poor EEG signals or malfunctions of the tACS stimulator to trigger intended electrical stimulation. The data of the remaining 31 participants were used for subsequent analysis (18 females, mean age ± SD: 22.8 ± 2.5 years, mean education ± SD: 16.2 ± 1.9 years). All participants gave written informed consent prior to the study.

## 8.2. Experimental procedures, stimulation paradigm, and EEG analysis

Each participant underwent three experimental sessions separated by at least 3 days at approximately the same time of the day. Using a single-blind within-subject design, participants received in-phase tACS (0° relative phase difference to alpha oscillations at Pz electrode), anti-phase tACS (180° relative phase difference to alpha oscillations at Pz electrode), or sham stimulation in each session, with the order of tACS condition counterbalanced across participants. The experimental procedure within each session and tACS parameters were the same as Experiment 1, except for the difficulty levels of the Sternberg task and the choice of sham stimulation as the control condition. In Experiment 2, trials were divided into two difficulty levels according to cognitive demands: participants were asked to remember seven consonants in half of the trials (“7-letter trials”) and asked to remember five consonants in the other half (“5-letter trials”). There were 30 5-letter trials and 30 7-letter trials at Baseline, 90 5-letter trials and 90 7-letter trials during Online tACS, and 30 5-letter trials and 30 7-letter trials during Offline tACS. The 5-letter trials and 7-letter trials were presented randomly. In the sham condition, to prevent participants from distinguishing between sham condition and active stimulation conditions, 0.8 s tACS trains were applied at the first trial of each set consisting of 60 trials during Online tACS.

In Experiment 2, the EEG data at Baseline and Online tACS were analyzed with the same methods as Experiment 1, except that the trials were divided into two types. The number of trials remaining after EEG-preprocessing was the same across the three tACS conditions. For every participant, in the two conditions with more trials, the shortest trials were deleted to make the number of trials consistent with the condition with the least trials. For the 5-letter trials, the mean and standard deviation of trial numbers across participants for Baseline and Online tACS were 25.71 ± 1.96 (range, 19-29) and 75.75 ± 4.81 (range, 60-89) respectively. For the 7-letter trials, the mean and standard deviation of trial numbers across participants for Baseline and Online tACS were 23.29 ± 2.62 (range, 15-29) and 74.38 ± 6.91 (range, 52-93) respectively.

**8.3. The 7-letter trials were difficult than 5-letter trials**

To examine the difference in difficulty between 5-letter trials and 7-letter trials, Baseline original RCS values were first analyzed using permuted two-way repeated-measures ANOVAs, with trial type (5-letter trials or 7-letter trials) and tACS condition (in-phase tACS or anti-phase tACS) as the within-subjects factors. We observed a significant main effect of trial type on RCS (F(1,30) = 88.445, p < 0.001, *ɳ*_p_^2^ = 0.747) (**S11 Fig**). Post-hoc permuted paired *t*-tests revealed that the Baseline RCS values in the 7-letter trials were significantly lower than those in the 5-letter trials in both in-phase condition (t(30) = -8.793, p < 0.001, Cohen’s d = 1.58) and anti-phase condition (t(30) = -5.829, p < 0.001, Cohen’s d = 1.05), demonstrating that participants made fewer correct responses per second of activity in the 7-letter trials.

## 8.4. The effects of sham condition were intermediate between the in-phase tACS and anti-phase tACS

To explore the effects of the sham condition, permuted two-way repeated-measures analysis of variance (ANOVA) were performed for behavioral performance and electrophysiological metrics, with trial type (5-letter trials or 7-letter trials) and tACS condition (in-phase tACS, sham stimulation, or anti-phase tACS) as within-subjects factors. During Online tACS, the main effect of tACS condition was significant for RCS (F(2,60) = 3.398, p = 0.036) (**S12A Fig**), with the effects of sham condition intermediate between the in-phase tACS and anti-phase tACS. The parietal alpha power and frontoparietal alpha synchronization in the sham condition were also intermediate between the in-phase tACS and anti-phase tACS, although the main effect of tACS condition was not significant (parietal alpha power: F(2,60) = 2.428, p = 0.100; frontoparietal alpha synchronization: F(2,60) = 1.043, p = 0.356) (**S12B and S12C Fig**). Permuted paired *t*-tests revealed that the differences between the sham condition and the two active tACS conditions were not significant for the three metrics (all p values were greater than 0.05).

## 8.5. Sham stimulation caused the problem of blind breaking

In the sham condition, 0.8 s tACS trains were only applied at the first trial of each set consisting of 60 trials during Online tACS. To examine whether participants could distinguish between sham condition and real stimulation conditions, participants were asked to report on how often they could feel themselves being stimulated (5-point Likert scale) and whether they believed they received real or sham stimulation (dichotomous response). As the frequency of feeling stimulated and the assessment of stimulation type are ordinal variables, we used Wilcoxon signed-rank tests for within-group comparisons. The “sham” answers in the sham condition significantly outweighed those in the real stimulation conditions (sham vs in-phase: z =2.111, p = 0.035; sham vs anti-phase: z = 2.496, p = 0.013; Wilcoxon signed-rank tests). The blind breaking may be because participants could find that they received tACS trains less frequently in the sham condition as compared to the two verum conditions. Compared to in-phase tACS or anti-phase tACS, participants in the sham condition reported feeling being stimulated less frequently (sham vs in-phase: z = -2.661, p = 0.008; sham vs anti-phase: z = -2.020, p = 0.043; Wilcoxon signed-rank tests). These results all suggest that participants could distinguish from sham condition and the real stimulation conditions.

Participants in Experiment 2 did not distinguish between the in-phase tACS and anti-phase tACS. There were no differences in participants “sham” answers (z = -1, p = 0.317; Wilcoxon signed-rank test) and the frequency of feeling stimulated (z = -0.836, p = 0.403; Wilcoxon signed-rank test) between in-phase tACS and anti-phase tACS. Besides, no differences in the general discomfort were observed between the in-phase tACS and anti-phase tACS (t(30) = -1.325, p = 0.219; permuted paired *t*-test).

# 9. **The phase-dependent tACS effects were detected from the combined data of Experiment 1 and Experiment 2**

As Experiment 1 and the 7-letter trials of Experiment 2 were identical in the experimental designs for the in-phase tACS and for anti-phase tACS, the data from Experiment 1 and the data from the 7-letter trials of Experiment 2 were combined to enlarge the sample size. We found that anti-phase tACS significantly decreased WM performance (t(69) = -3.811, p < 0.001, Cohen’s d = 0.456), parietal alpha oscillations (t(69) = -3.101, p = 0.004, Cohen’s d = 0.371), and frontoparietal alpha synchronization (t(69) = -2.377, p = 0.020, Cohen’s d = 0.284). These results support that the phase differences between tACS signals and targeted brain oscillations influence tACS effects.

# 10. Experiment 3

## 10.1. Participants

44 volunteers participated in Experiment 3. One participant was excluded from the analyses due to equipment failure to trigger intended electrical stimulation. The data of the remaining 43 participants were included for subsequent analysis (28 females, mean age ± SD：22.6 ± 2.1 years, mean education ± SD：16.4 ± 1.9 years). All participants gave written informed consent prior to the study.

## 10.2. Experimental procedures, stimulation paradigm, and EEG analysis

Our choice of theta tACS as a control was motivated by multiple EEG and MEG studies suggesting that local frontal—rather than parietal—theta oscillations are related to WM retention in the Sternberg task [10]. Parietal-occipital theta power during the retention sub-process of the Sternberg task does not increase for the memory condition when compared to the control condition [10], and does not increase with memory difficulty [10-12].

Each participant underwent 2 experimental sessions separated by at least 3 days at approximately the same time of the day. Using a single-blind within-subject design, participants received in-phase tACS (0° relative phase difference to theta oscillations at Pz electrode) or anti-phase tACS (180° relative phase difference to theta oscillations at Pz electrode) in each session, with the order of sessions counterbalanced across participants. The experimental procedure within each session was the same as Experiment 1, including the first 60 trials (Baseline), the next 180 tACS-EEG trials (Online tACS), and the final 60 trials (Offline tACS).

The stimulation parameters of Experiment 3 were the same as Experiment 1 except for tACS frequency and the threshold calculation. After the first 60 EEG trials (Baseline), IAF was calculated using the EEG signal at Baseline. Individual theta frequency (ITF) was then determined as (IAF – 5) Hz as some previous studies did [13]. During Online tACS, our online phase-corrected tACS system was applied to deliver tACS at ITF over parietal-occipital cortex when EEG signals met requirements. For threshold calculation, the 2.5% quantile rather than the lower quartile of all peaks or all throughs was used as the threshold for triggering tACS. Due to the weak parietal theta oscillations and the fewer cycles of theta oscillations, the lower threshold for theta tACS was set to make the stimulation numbers of theta tACS comparable to alpha tACS.

In Experiment 3, parietal theta power (3-8 Hz) at Baseline and Online tACS were analyzed with the same methods as Experiment 1, except that the frequency band of spectra analysis was changed to theta frequency band. After EEG pre-processing, the number of trials was the same between in-phase tACS and anti-phase tACS, as we matched the number of trials across tACS conditions for every participant. The mean and standard deviation of trial numbers across participants for Baseline and Online tACS were 48.86 ± 4.20 (range, 39-55) and 144.91 ± 11.70 (range, 115-167) respectively.

## 10.3. The effects of theta tACS differed from alpha tACS

To test whether the effects induced by parietal theta tACS differed from the effects of parietal alpha tACS, we have now performed a permuted two-way mixed ANOVA on behavioral performance (i.e., RCS) with tACS frequency (alpha frequency versus theta frequency) as the between-subjects factor and the tACS condition (in-phase tACS versus anti-phase tACS) as the within-subjects factor. Both Experiment 1 and the 7-letter trials of Experiment 2 applied tACS at the alpha frequency, and they were identical in the experimental designs for in-phase tACS and for anti-phase tACS. To enlarger the sample size, the data from Experiment 1 and the data from the 7-letter trials of Experiment 2 were combined together to assess the effects induced by alpha tACS. We found a significant frequency × condition interaction (F(1, 111) = 4.025, p = 0.041, *ɳ*_p_^2^ = 0.035; permuted two-way mixed ANOVA), supporting that alpha tACS and theta tACS lead to different behavioral outcomes.

## 10.4. Blinding

Theta-tACS induced discomfort were computed using the method same as Experiment 1. There were no differences in the general discomfort between the tACS conditions (in-phase vs anti-phase: t(42) = 0.151, p = 1). This also demonstrated that participants were not able to distinguish between in-phase tACS and anti-phase tACS in Experiment 3.

# 11. Experiment 4

## 11.1. Participants

20 volunteers participated in Experiment 4. Data from all 20 participants were included for subsequent behavioral and EEG analyses (8 females, mean age ± SD：22.1 ± 2.1 years, mean education ± SD：15.7 ± 2.1 years). All participants gave written informed consent prior to the study.

## 11.2. Experimental procedures, stimulation paradigm, and EEG analysis

Each participant underwent 2 experimental sessions separated by at least 3 days at approximately the same time of the day. Using a single-blind within-subject design, participants received in-phase tACS or anti-phase tACS in each session, with the order of tACS condition counterbalanced across participants. The experimental procedure within each session was the same as Experiment 1, including the first 60 trials (Baseline), the next 180 tACS-EEG trials (Online tACS), and the final 60 trials (Offline tACS).

The tACS parameters were also the same as Experiment 1 except for the position of the stimulation electrode. The stimulation electrode (4 * 6 cm) was placed at the vertex region (i.e., Cz electrode) due to the following two reasons: (1) as the vertex region is thought to be irrelevant to WM and has been used as a control site in previous WM-related brain stimulation studies [14, 15], Experiment 4 excluded the effects of cortical stimulation; (2) as the retina is relatively closer to the vertex region rather than the parietal cortex, montages in Experiment 4 can be assumed to produce equivalent or stronger retinal effects than Experiment 1 [16]. IAF was calculated using the EEG signals at the Pz electrode measured at Baseline. During Online tACS, in-phase tACS (0° relative phase difference to alpha oscillations at Pz electrode) or anti-phase tACS (180° relative phase difference to alpha oscillations at Pz electrode) at IAF was applied to the vertex.

In Experiment 4, parietal alpha power at Baseline and Online tACS were analyzed with the same methods as Experiment 1. After EEG pre-processing, the number of trials was the same between in-phase tACS and anti-phase tACS, as we matched the number of trials across tACS conditions for every participant. The mean and standard deviation of trial numbers across participants for Baseline and Online tACS were 50.48 ± 4.41 (range, 39-56) and 148.48 ± 11.46 (range, 115-168) respectively.

## 11.3. Blinding

tACS induced sensations were computed using the same method described in Experiment 1. There were also no differences in the perceived discomfort between tACS conditions (in-phase vs anti-phase: t(19) = -0.890, p = 0.495; permuted paired *t*-test). This result also demonstrated that participants were not able to distinguish in-phase tACS and anti-phase tACS in Experiment 4.

# 12. Supplementary discussion

## 12.1. A discussion regarding positioning of the stimulation electrode

The stimulation electrode in our study was not precisely located at the monitored Pz electrode, but between the Pz electrode and the Oz electrode to avoid the pollution of EEG signals. One may wonder whether tACS in our study was able to target the alpha activity recorded from Pz electrode. Alpha activity recorded from Pz electrode generated from the widely distributed sources in parieto-occipital regions [17], supporting the capacity of our tACS to target these sources.

## 12.2. A discussion on the non-significance of in-phase tACS-induced enhancement effects

The improvement effects on WM performance and alpha activity induced by in-phase tACS were not significant during Online tACS, so that one may question the enhancement effects of in-phase tACS. Please note that although not reaching the significance, in-phase tACS showed the tendency to enhance all of the three detected metrics (i.e., RCS, parietal alpha power and frontal-parietal alpha synchronization) during tACS. The lack of significant enhancement may be due to the fact that it is difficult for tACS to increase endogenous so strong alpha oscillations [18] induced by the difficult Sternberg paradigm.

**References**

1. Jones AP, Choe J, Bryant NB, Robinson CSH, Ketz NA, Skorheim SW, et al. Dose-Dependent Effects of Closed-Loop tACS Delivered During Slow-Wave Oscillations on Memory Consolidation. Front Neurosci. 2018;12. doi: 10.3389/fnins.2018.00867.

2. Ketz N, Jones AP, Bryant NB, Clark VP, Pilly PK. Closed-Loop Slow-Wave tACS Improves Sleep-Dependent Long-Term Memory Generalization by Modulating Endogenous Oscillations. J Neurosci. 2018;38(33):7314-26. doi: 10.1523/jneurosci.0273-18.2018.

3. Lustenberger C, Boyle MR, Alagapan S, Mellin JM, Vaughn BV, Frohlich F. Feedback-Controlled Transcranial Alternating Current Stimulation Reveals a Functional Role of Sleep Spindles in Motor Memory Consolidation. Curr Biol. 2016;26(16):2127-36. doi: 10.1016/j.cub.2016.06.044.

4. Benwell CSY, London RE, Tagliabue CF, Veniero D, Gross J, Keitel C, et al. Frequency and power of human alpha oscillations drift systematically with time-on-task. Neuroimage. 2019;192:101-14. doi: 10.1016/j.neuroimage.2019.02.067.

5. Berens P. CircStat: a MATLAB toolbox for circular statistics. J Stat Softw. 2009;31(10):1-21.

6. Froehlich F, McCormick DA. Endogenous Electric Fields May Guide Neocortical Network Activity. Neuron. 2010;67(1):129-43. doi: 10.1016/j.neuron.2010.06.005.

7. Zaehle T, Rach S, Herrmann CS. Transcranial Alternating Current Stimulation Enhances Individual Alpha Activity in Human EEG. Plos One. 2010;5(11). doi: 10.1371/journal.pone.0013766.

8. Vinck M, Oostenveld R, Van Wingerden M, Battaglia F, Pennartz CM. An improved index of phase-synchronization for electrophysiological data in the presence of volume-conduction, noise and sample-size bias. Neuroimage. 2011;55(4):1548-65.

9. Gevins A, Smith ME, McEvoy L, Yu D. High-resolution EEG mapping of cortical activation related to working memory: Effects of task difficulty, type of processing, and practice. Cereb Cortex. 1997;7(4):374-85. doi: 10.1093/cercor/7.4.374.

10. Jensen O, Tesche CD. Frontal theta activity in humans increases with memory load in a working memory task. Eur J Neurosci. 2002;15(8):1395-9.

11. Jensen O, Gelfand J, Kounios J, Lisman JE. Oscillations in the alpha band (9-12 Hz) increase with memory load during retention in a short-term memory task. Cereb Cortex. 2002;12(8):877-82. doi: 10.1093/cercor/12.8.877.

12. Zhang D, Zhao H, Bai W, Tian X. Functional connectivity among multi-channel EEGs when working memory load reaches the capacity. Brain Res. 2016;1631:101-12. doi: 10.1016/j.brainres.2015.11.036.

13. Jausovec N, Jausovec K. Increasing working memory capacity with theta transcranial alternating current stimulation (tACS). Biol Psychol. 2014;96:42-7. doi: 10.1016/j.biopsycho.2013.11.006.

14. Fried PJ, Rushmore RJ, 3rd, Moss MB, Valero-Cabre A, Pascual-Leone A. Causal evidence supporting functional dissociation of verbal and spatial working memory in the human dorsolateral prefrontal cortex. Eur J Neurosci. 2014;39(11):1973-81. doi: 10.1111/ejn.12584.

15. Kiyonaga A, Korb FM, Lucas J, Soto D, Egner T. Dissociable causal roles for left and right parietal cortex in controlling attentional biases from the contents of working memory. Neuroimage. 2014;100:200-5. doi: 10.1016/j.neuroimage.2014.06.019.

16. Kar K, Krekelberg B. Transcranial electrical stimulation over visual cortex evokes phosphenes with a retinal origin. J Neurophysiol. 2012;108(8):2173-8. doi: 10.1152/jn.00505.2012.

17. Tuladhar AM, ter Huurne N, Schoffelen JM, Maris E, Oostenveld R, Jensen O. Parieto-occipital sources account for the increase in alpha activity with working memory load. Hum Brain Mapp. 2007;28(8):785-92. doi: 10.1002/hbm.20306.

18. Neuling T, Rach S, Herrmann CS. Orchestrating neuronal networks: sustained after-effects of transcranial alternating current stimulation depend upon brain states. Front Hum Neurosci. 2013;7. doi: 10.3389/fnhum.2013.00161.
